# Supplementary material for: Bacterial Volatiles (mVOC) Emitted by the Phytopathogen Erwinia amylovora Promote Arabidopsis thaliana Growth and Oxidative Stress
Source: Antioxidants (Basel). 2023 Feb 28;12(3):600. doi: 10.3390/antiox12030600 (PMC10045578; doi:10.3390/antiox12030600)

# Supplementary Figure S1

Seedlings of *A. thaliana* are cultivated in the presence of synthetic mVOCs. Arrow indicates the filter paper disk where the synthetic chemical is placed.

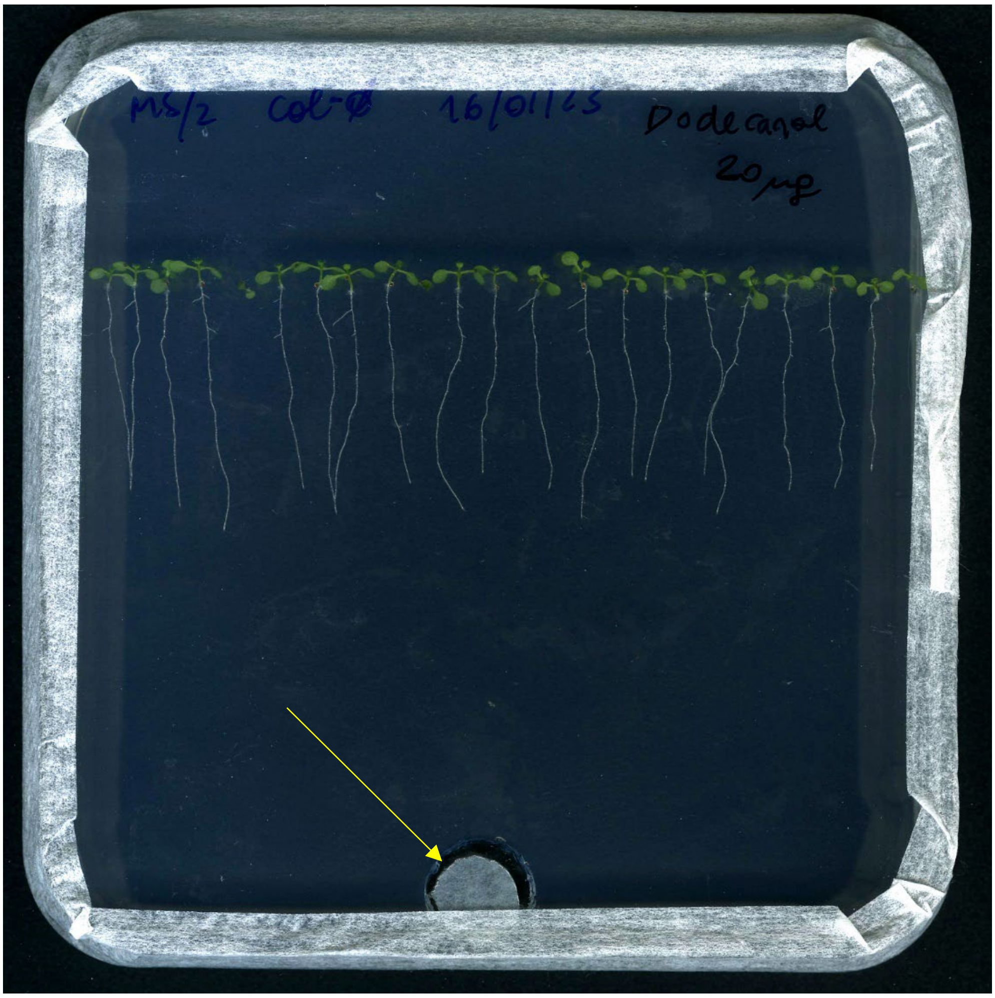

Supplement: Supplementary file 1 [file antioxidants-12-00600-s001.zip › Supplementary Figure S1.pdf]
