# Supplementary material for: Bacterial Volatiles (mVOC) Emitted by the Phytopathogen Erwinia amylovora Promote Arabidopsis thaliana Growth and Oxidative Stress
Source: Antioxidants (Basel). 2023 Feb 28;12(3):600. doi: 10.3390/antiox12030600 (PMC10045578; doi:10.3390/antiox12030600)
Supplement: Supplementary file 1 [file antioxidants-12-00600-s001.zip › Supplementary Figure S2.pdf]

Supplementary Figure S2 GC-MS Spectra data

dichloromethyl propyl sulfone

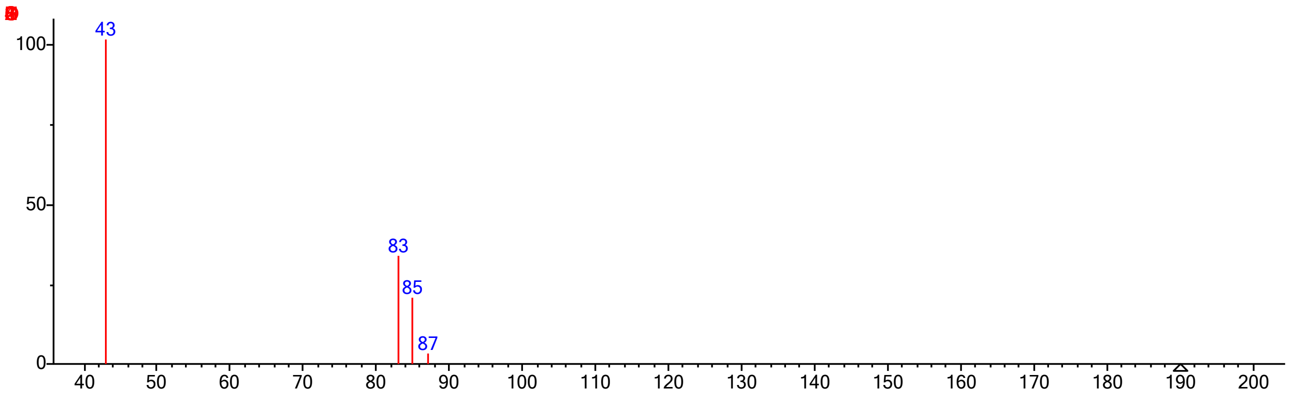

(wiley7) dichloromethyl propyl sulfone

2-Ethyl-1-decanol

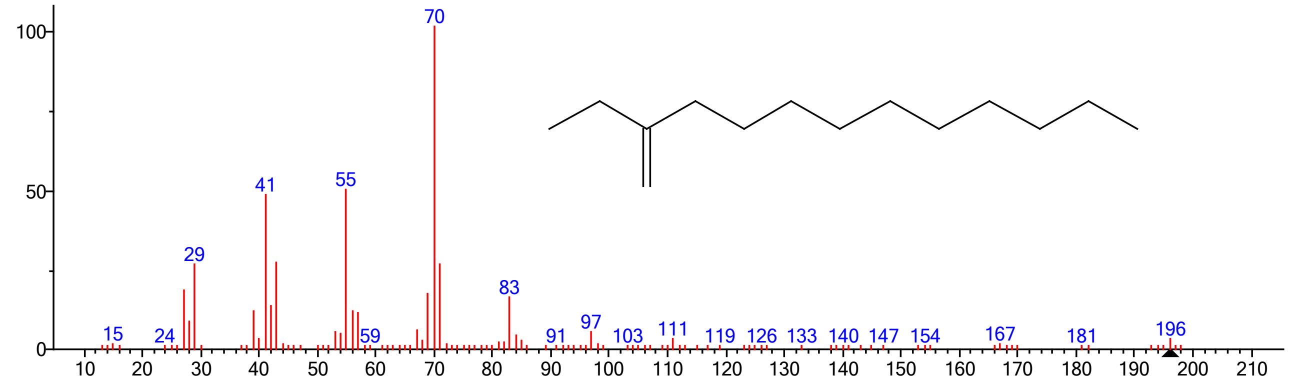

(wiley7) 1-Dodecene, 2-ethyl- (CAS)

1,8-Cineole

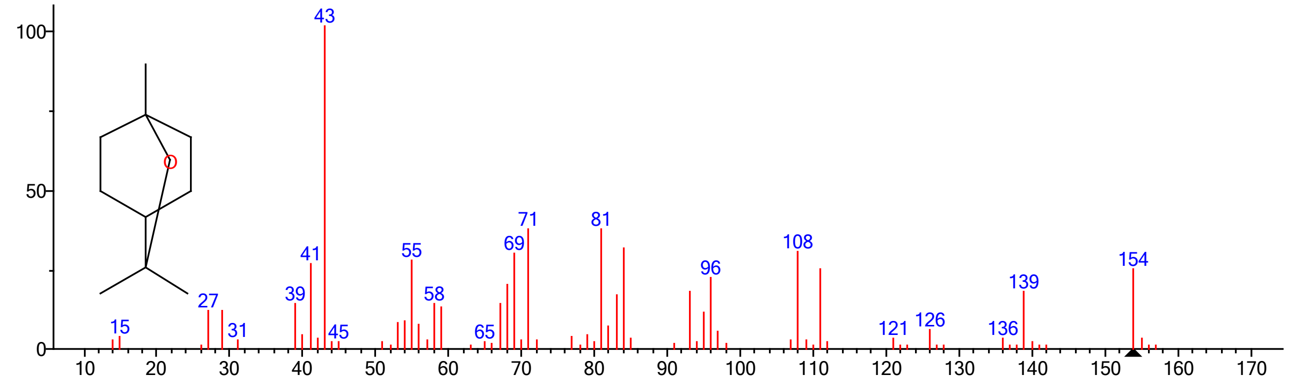

(wiley7) 1,8-Cineole

iso amyl alcohol

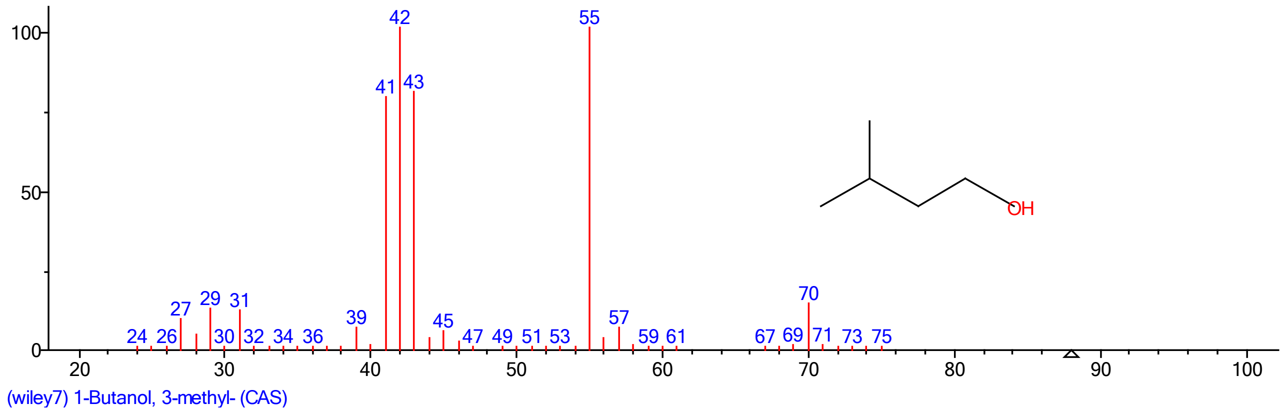

6-Methyl-2-heptanone

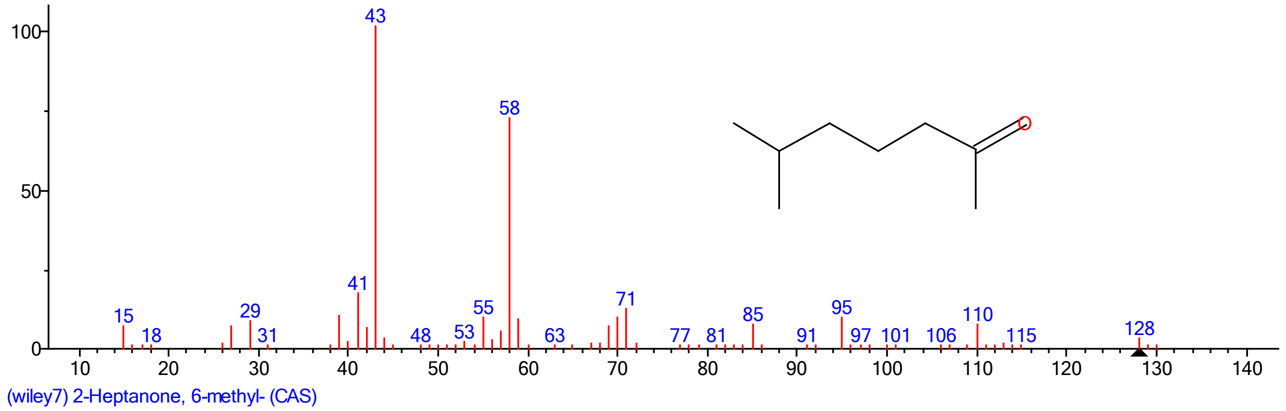

Styrene

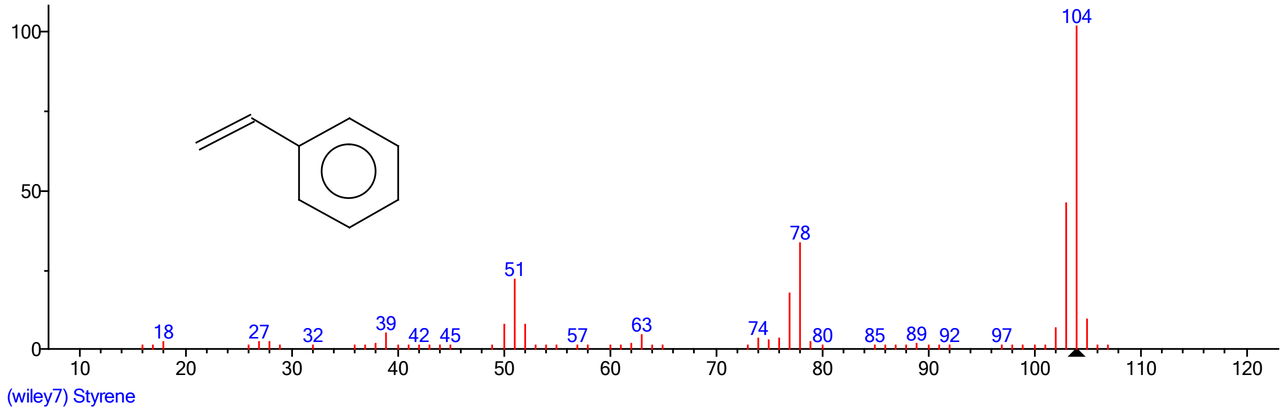

Octanal

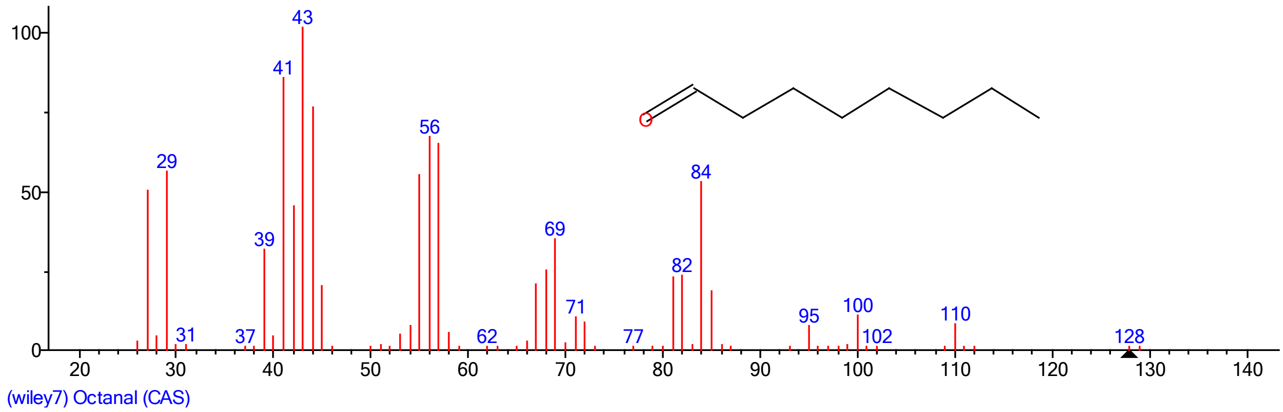

Tridecane

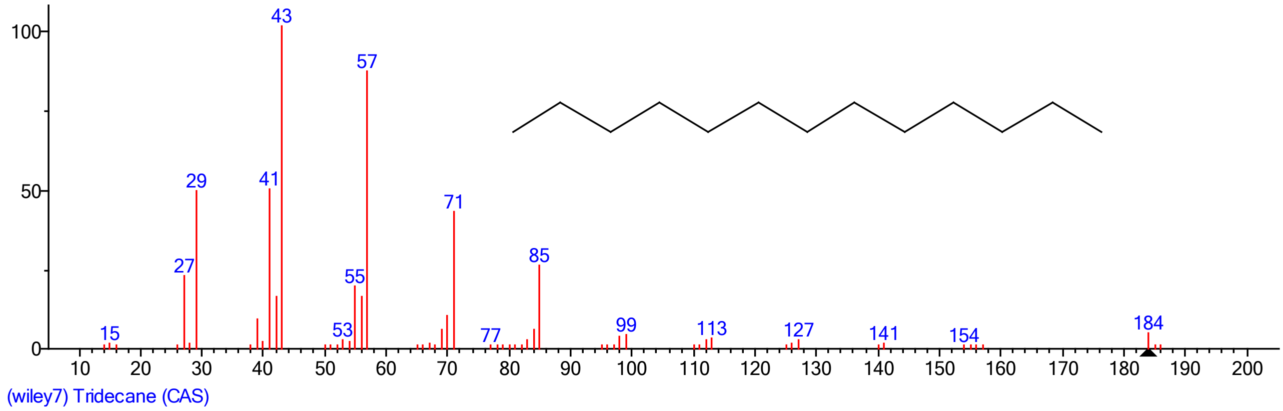

Tetraisobutylene

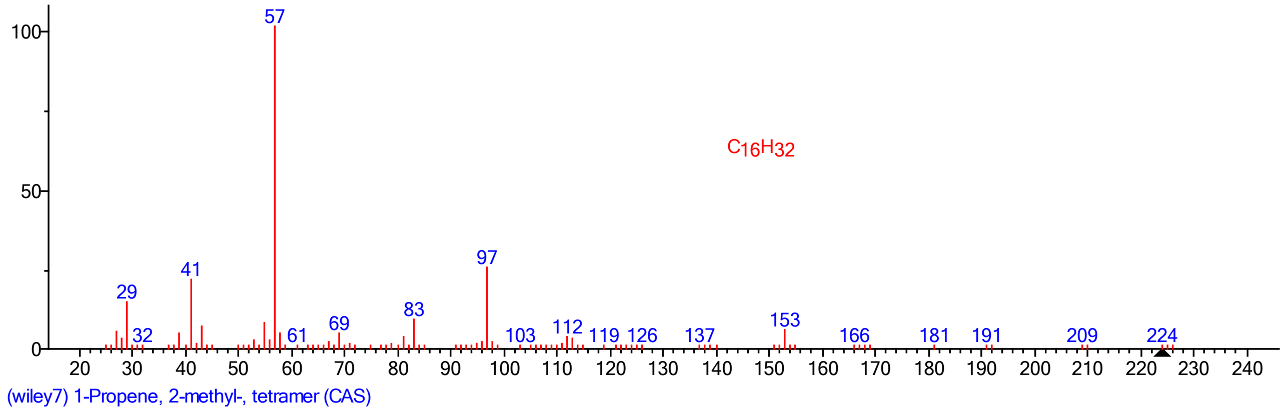

4-Octen-3-one

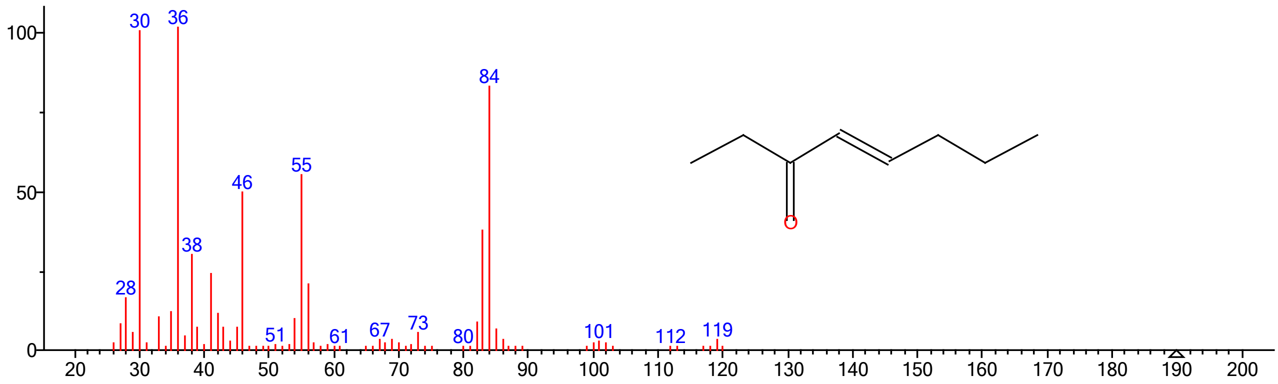

(wiley7) 4-Octen-3-one (CAS)

Nonanal

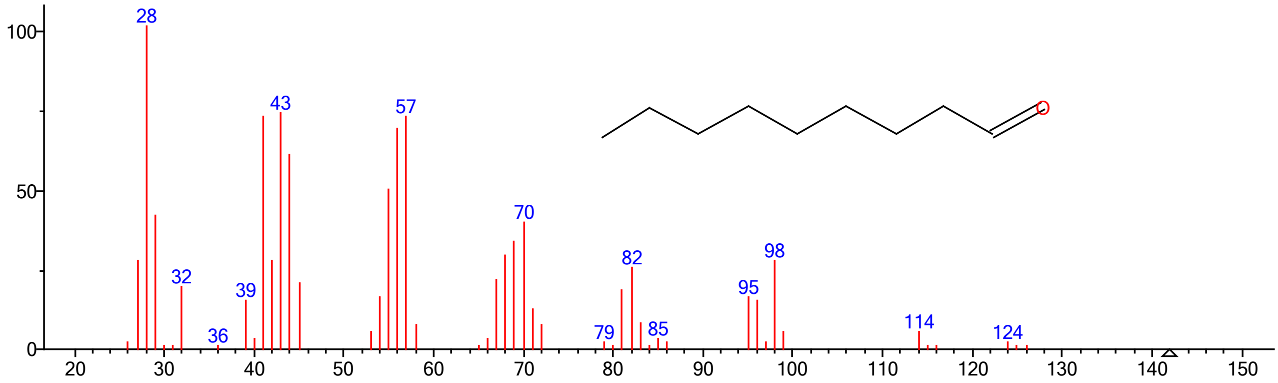

(wiley7) Nonanal (CAS)

Tetradecane

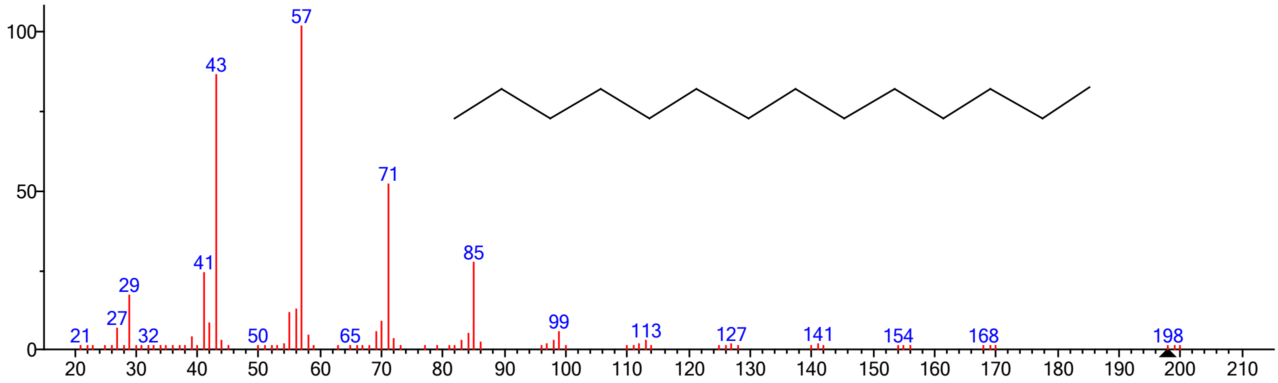

(wiley7) Tetradecane (CAS)

(E)-2-Octenal

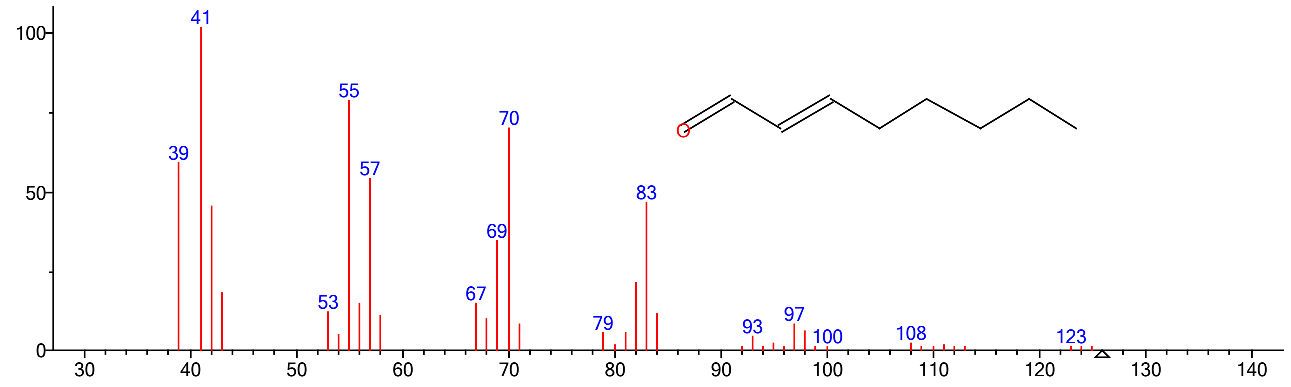

(wiley7) 2-Octenal, (E)- (CAS)

1-Tetradecene

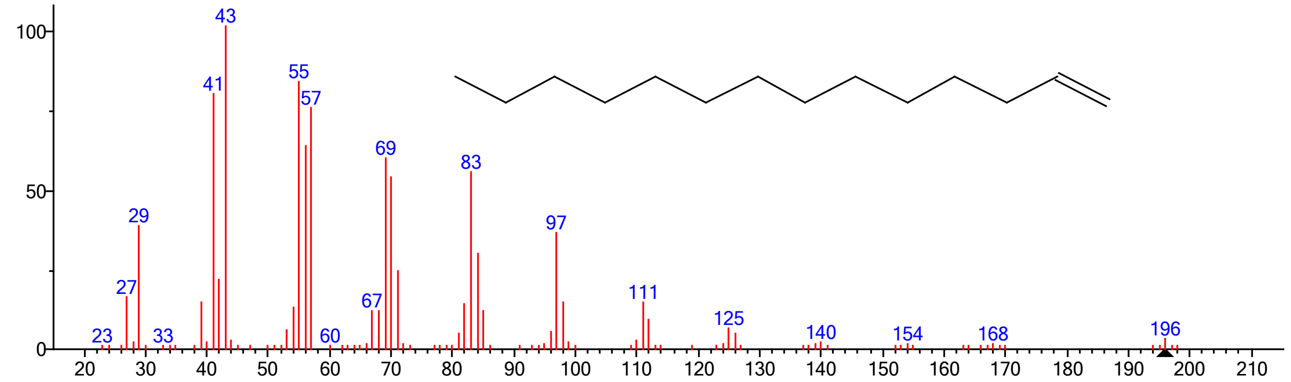

(wiley7) 1-Tetradecene (CAS)

Undecanal

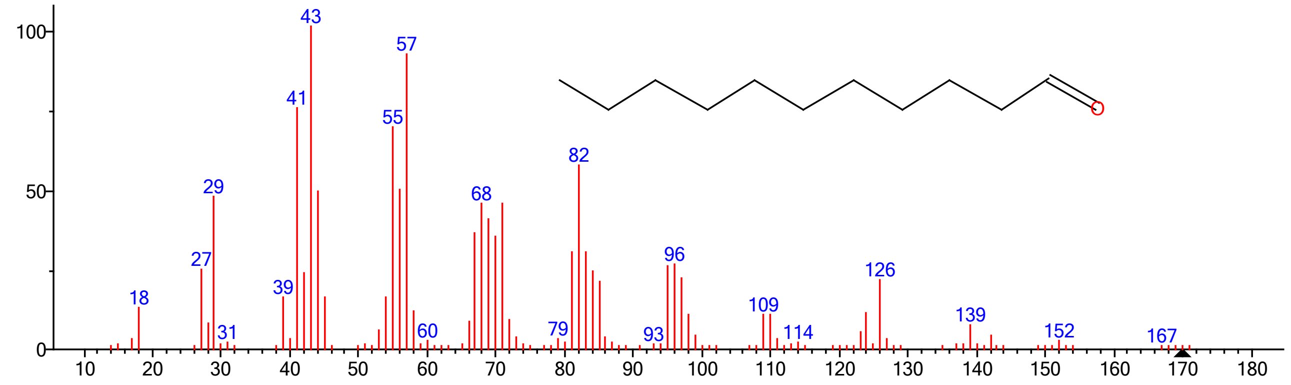

(wiley7) Undecanal (CAS)

Pentadecane

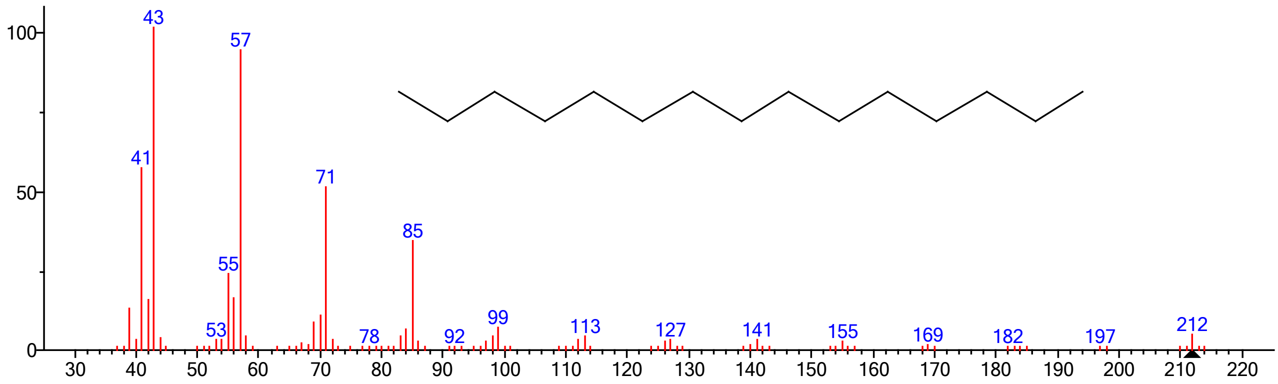

(wiley7) Pentadecane (CAS)

Benzaldehyde

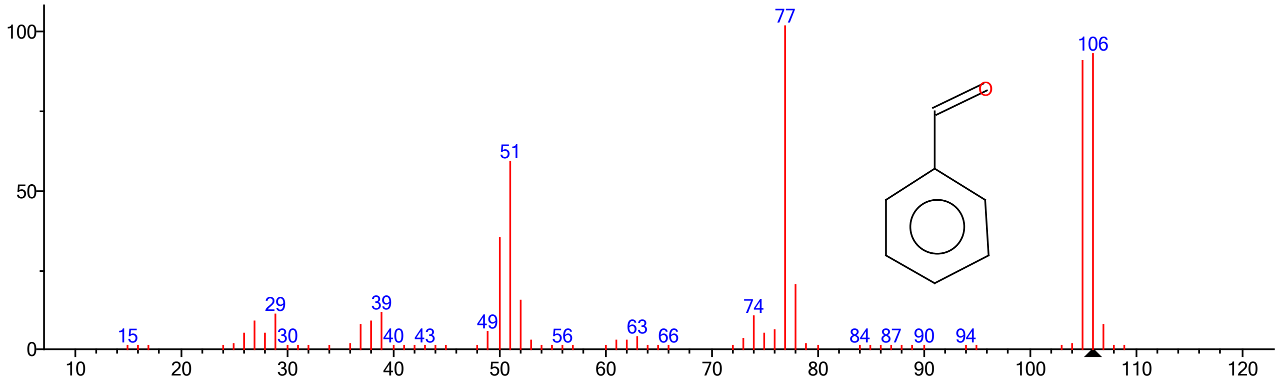

(wiley7) Benzaldehyde (CAS)

E-2-dodecenal

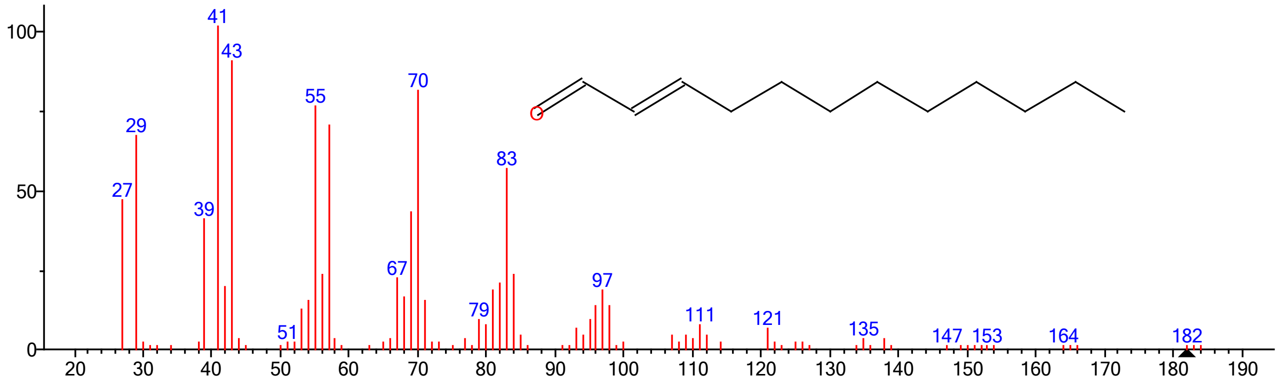

(wiley7) E-2-dodecenal

Tetradecanal

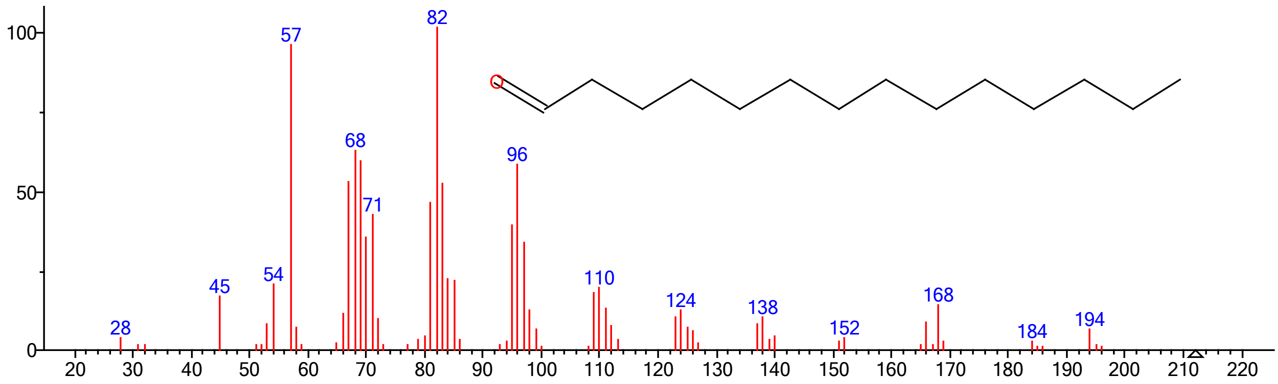

(wiley7) Tetradecanal (CAS)

trans-2-Decenal

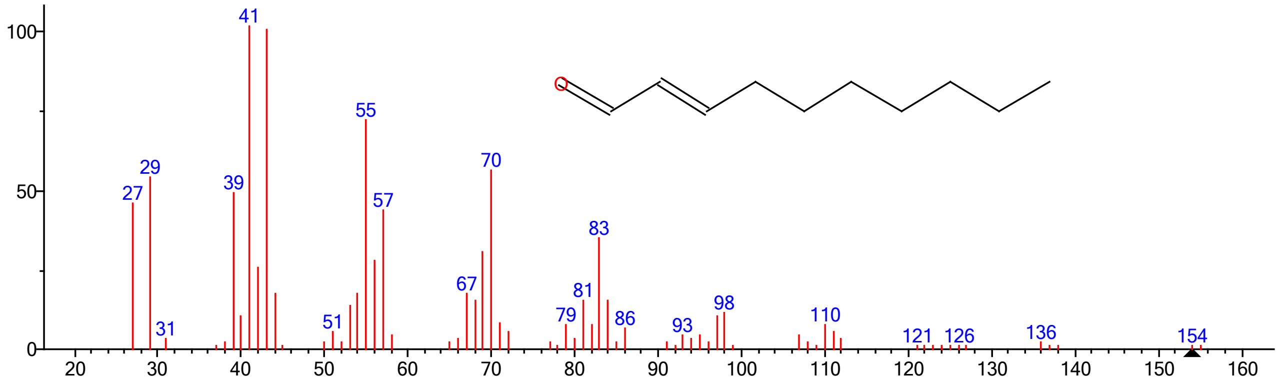

(wiley7) 2-Decenal, (E)- (CAS)

1-Nonanol

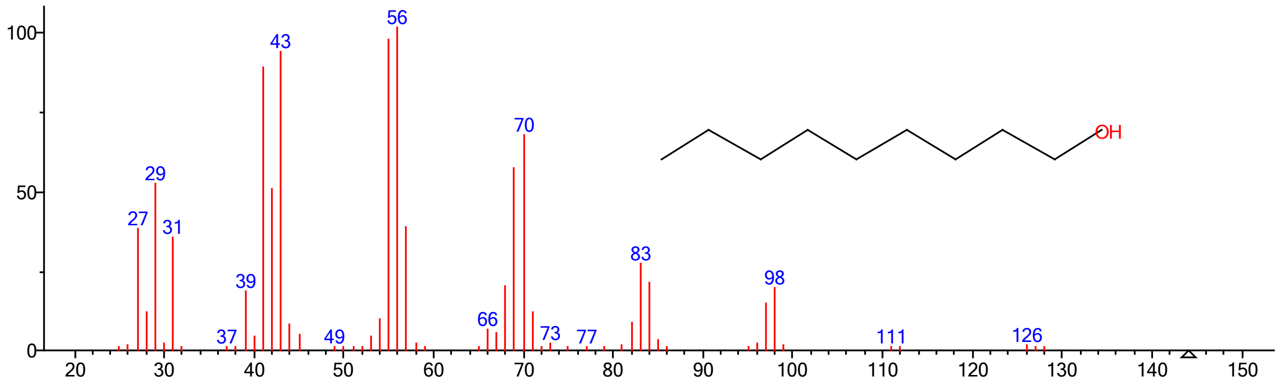

(wiley7) 1-Nonanol (CAS)

6,10-Dimethyl-2-undecanone

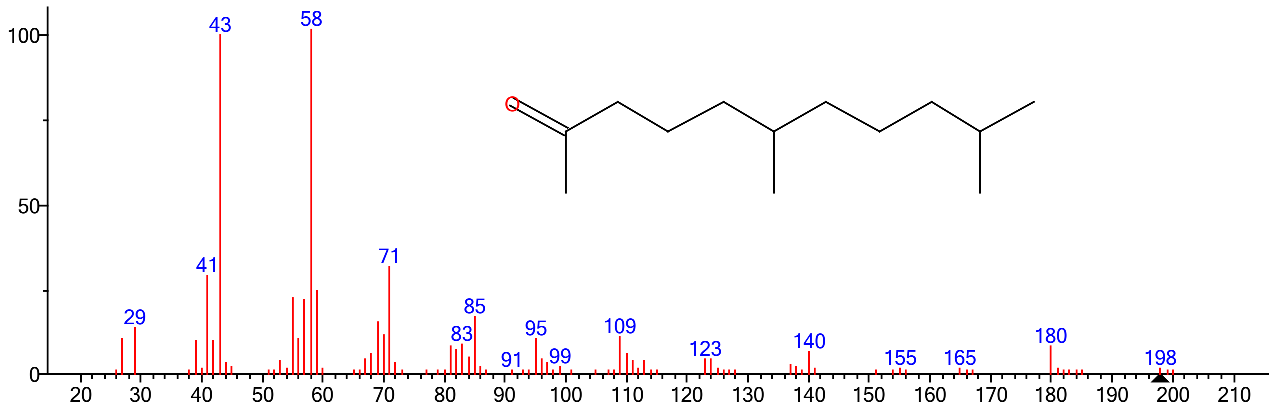

(wiley7) 2-Undecanone, 6,10-dimethyl- (CAS)

Heptadecane

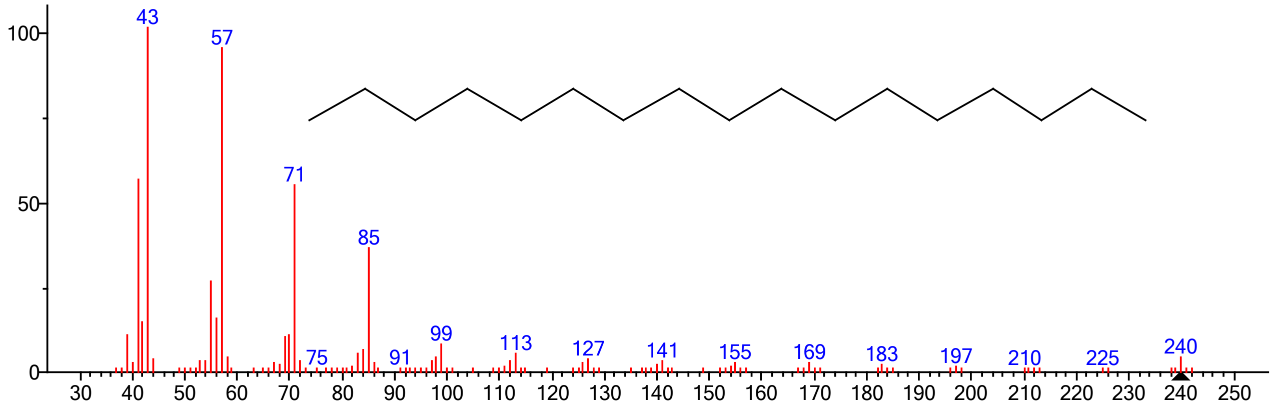

(wiley7) Heptadecane (CAS)

Methylcyclodecane

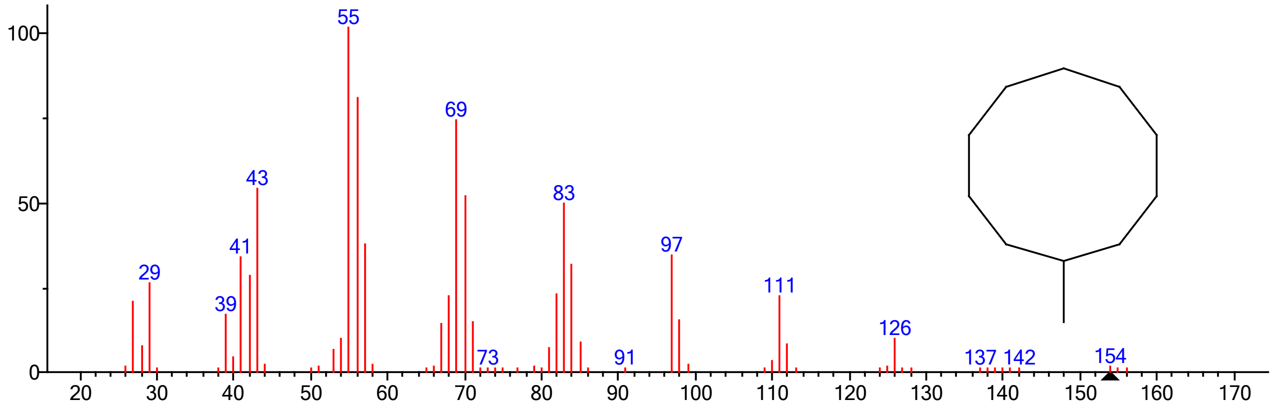

(wiley7) Cyclodecane, methyl- (CAS)

Tridecanal

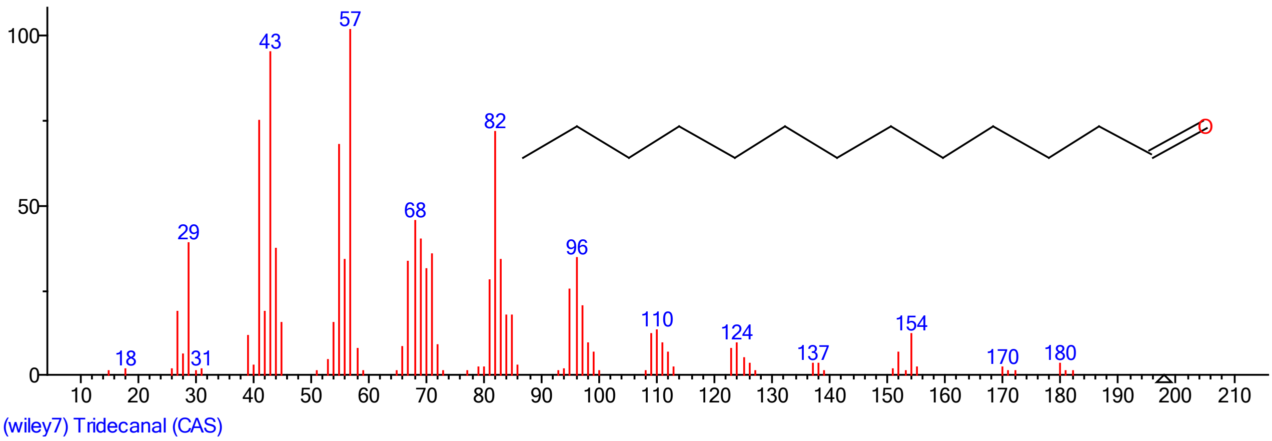

trans-2-Decenol

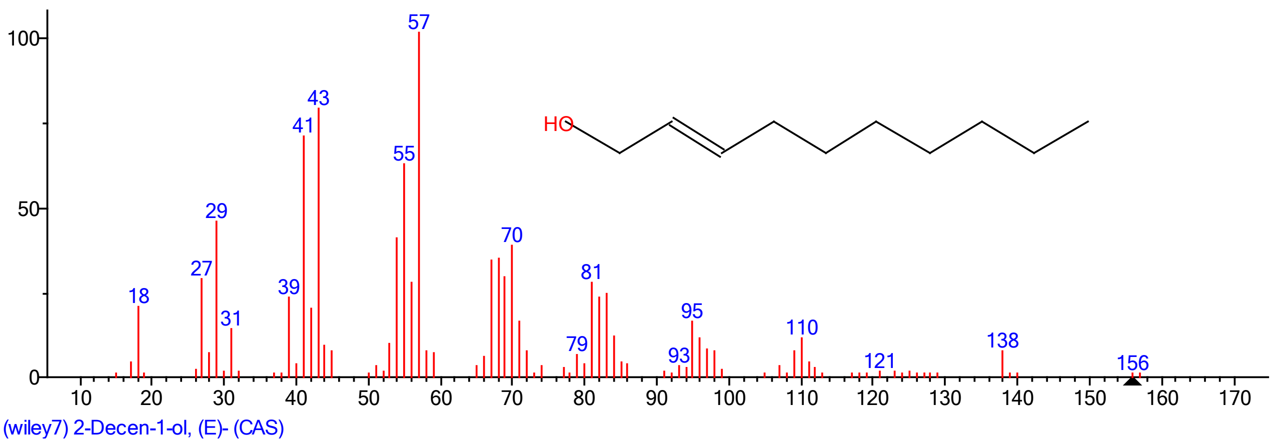

1-Undecanol

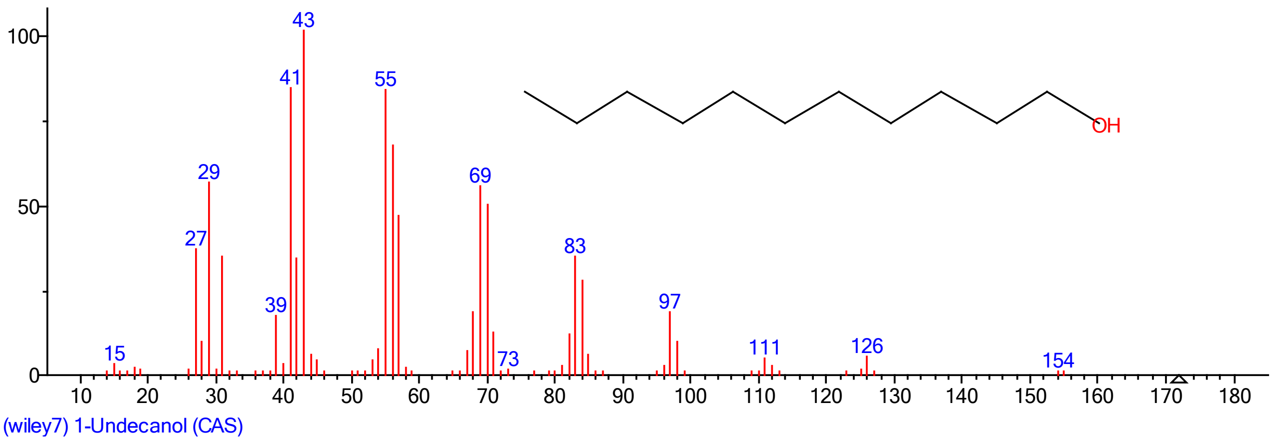

1-Dodecanol

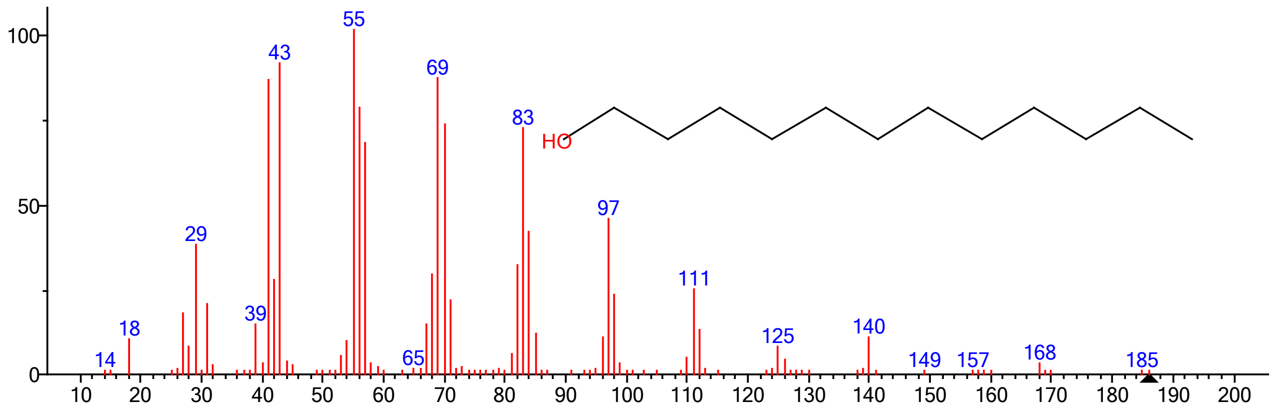

(wiley7) 1-Dodecanol (CAS)

1-Tetradecanol

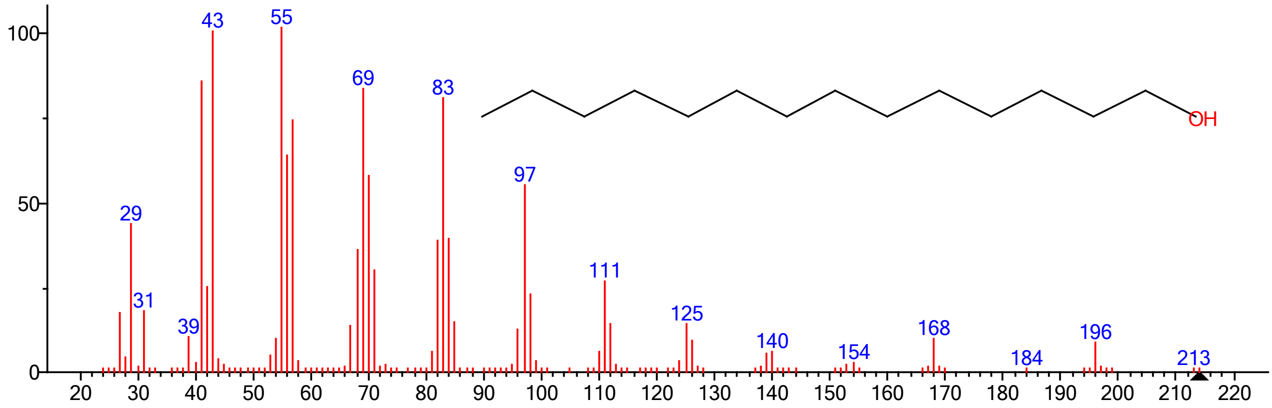

(wiley7) 1-Tetradecanol (CAS)

2-Pentadecanone

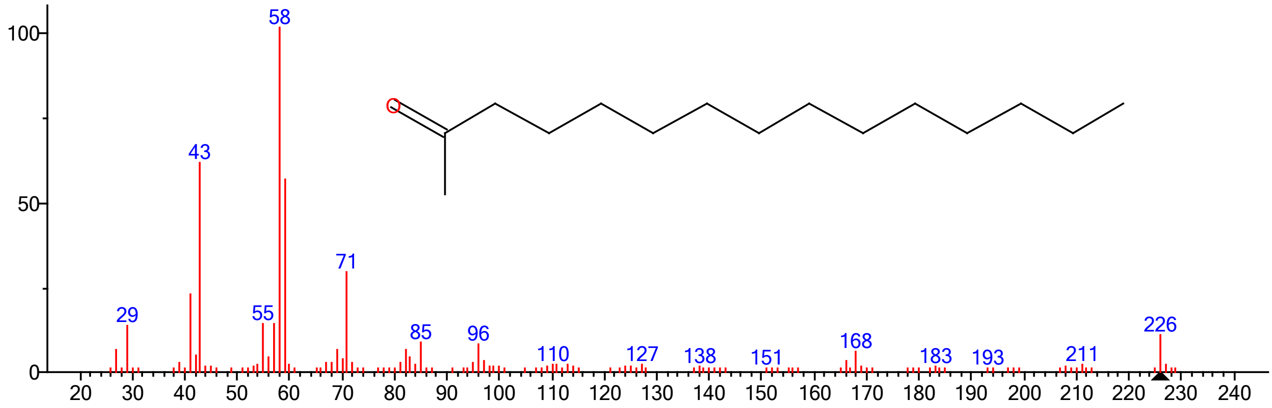

(wiley7) 2-Pentadecanone (CAS)

Pentadecanal

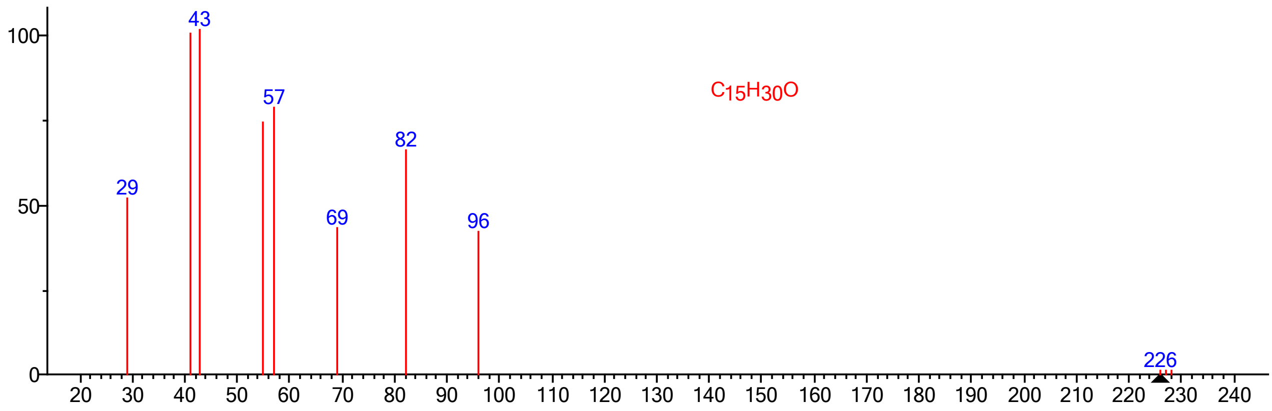

(wiley7) Pentadecanal

6,10,14-Trimethyl-2-pentadecanone

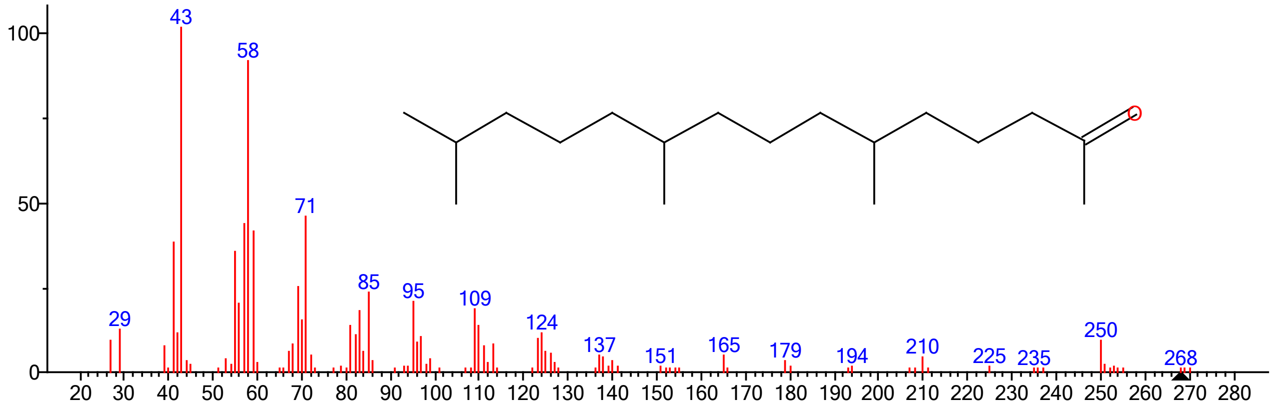

(wiley7) 2-Pentadecanone, 6,10,14-trimethyl- (CAS)

1-octanol

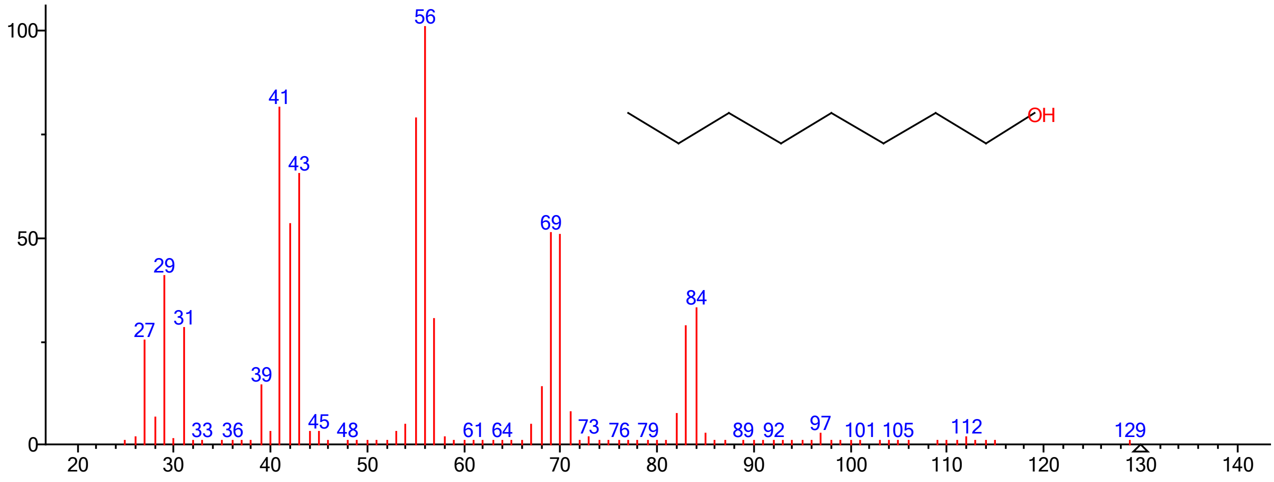

(wiley7) 1-Octanol (CAS)
