# Supplementary material for: Bacterial Volatiles (mVOC) Emitted by the Phytopathogen Erwinia amylovora Promote Arabidopsis thaliana Growth and Oxidative Stress
Source: Antioxidants (Basel). 2023 Feb 28;12(3):600. doi: 10.3390/antiox12030600 (PMC10045578; doi:10.3390/antiox12030600)
Supplement: Supplementary file 1 [file antioxidants-12-00600-s001.zip › Supplementary Figure S3.pdf]

Supplementary Figure S3 Functional Categorization

UPREGULATED GENES

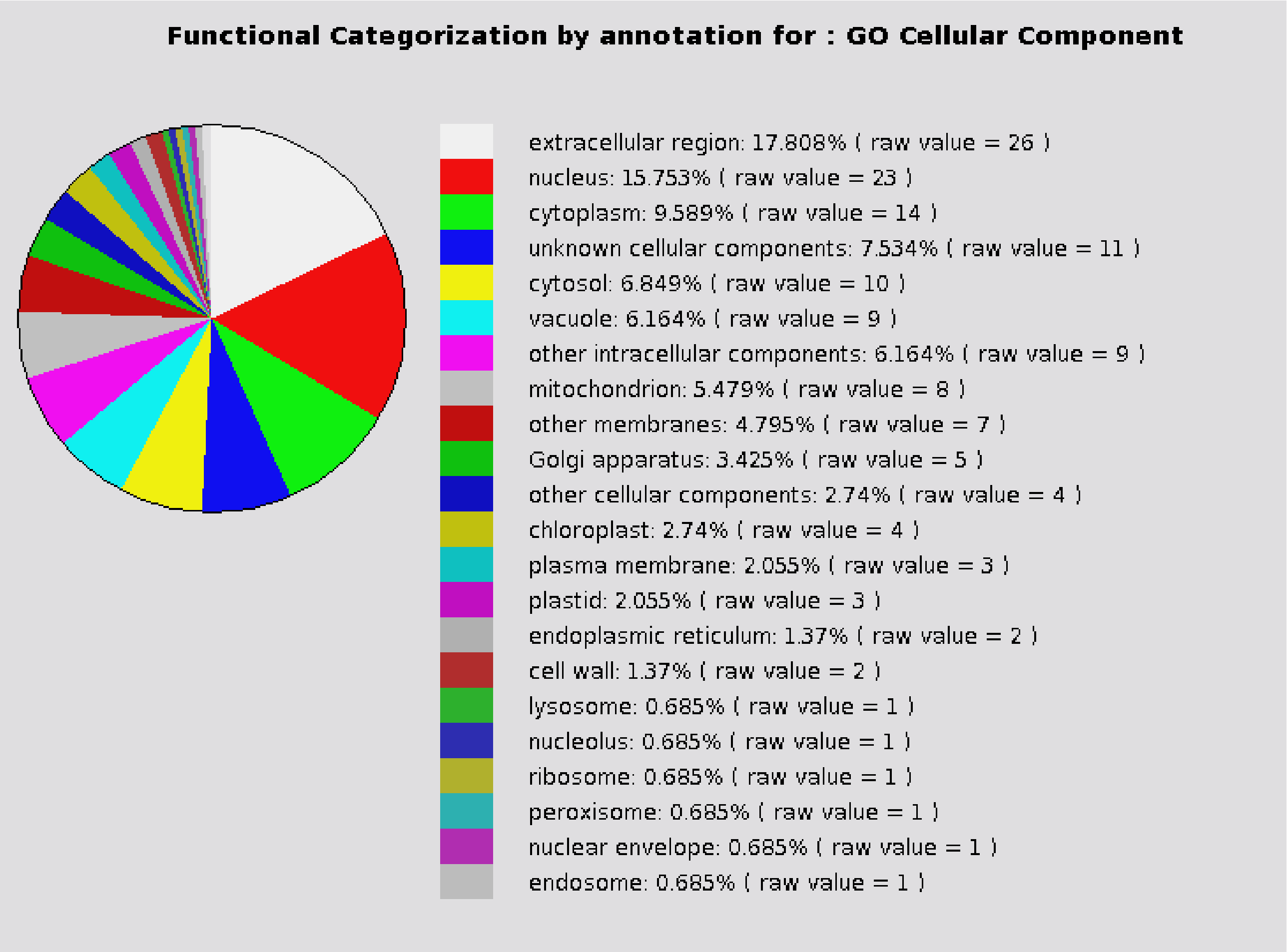

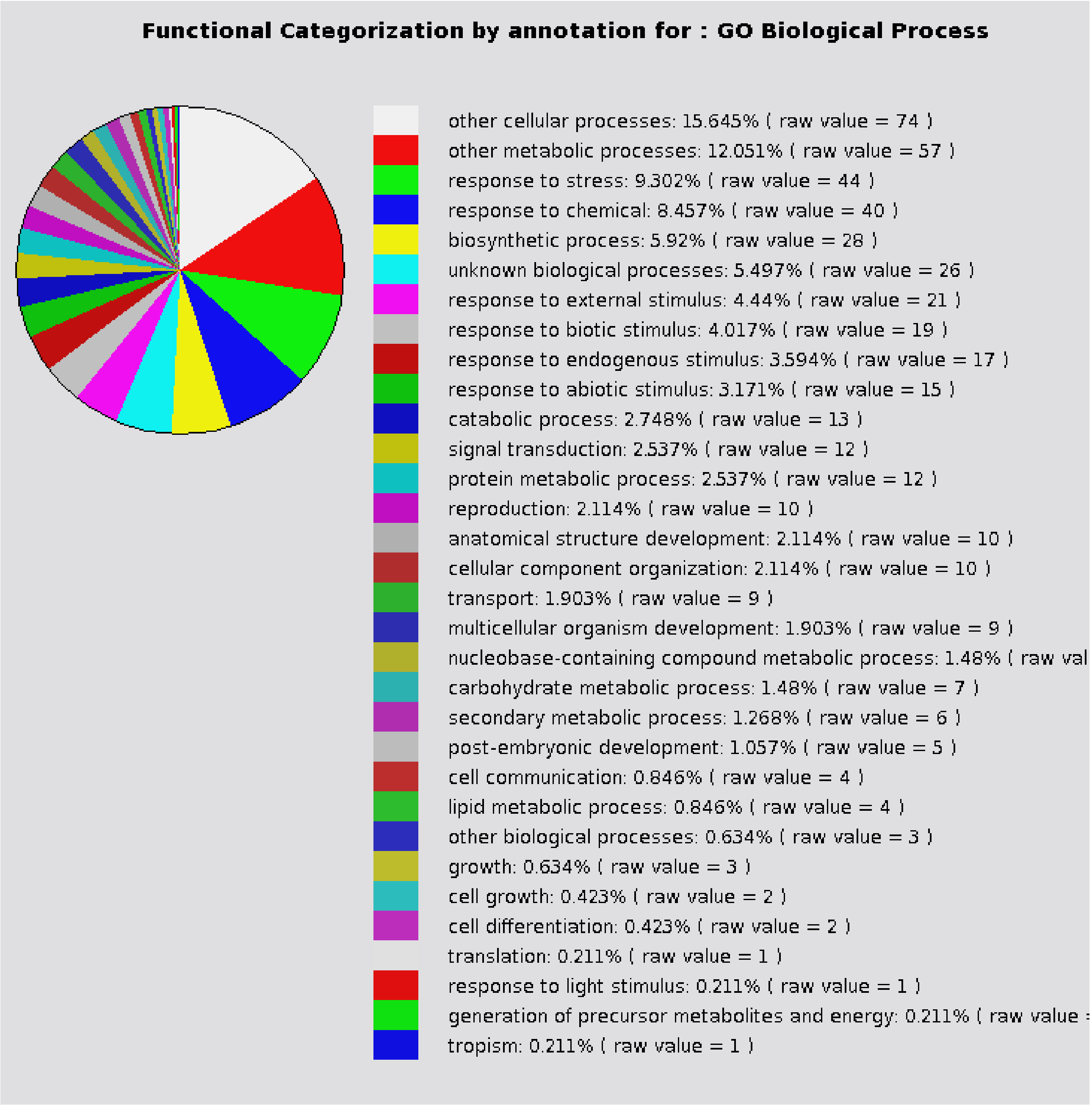
Functional Categorization by annotation for : GO Biological Process
other cellular processes: 15.645% (raw value = 74)
other metabolic processes: 12.051% (raw value = 57)
response to stress: 9.302% (raw value = 44)
response to chemical: 8.457% (raw value = 40)
biosynthetic process: 5.92% (raw value = 28)
unknown biological processes: 5.497% (raw value = 26)
response to external stimulus: 4.44% (raw value = 21)
response to biotic stimulus: 4.017% (raw value = 19)
response to endogenous stimulus: 3.594% (raw value = 17)
response to abiotic stimulus: 3.171% (raw value = 15)
catabolic process: 2.748% (raw value = 13)
signal transduction: 2.537% (raw value = 12)
protein metabolic process: 2.537% (raw value = 12)
reproduction: 2.114% (raw value = 10)
anatomical structure development: 2.114% (raw value = 10)
cellular component organization: 2.114% (raw value = 10)
transport: 1.903% (raw value = 9)
multicellular organism development: 1.903% (raw value = 9)
nucleobase-containing compound metabolic process: 1.48% (raw val
carbohydrate metabolic process: 1.48% (raw value = 7)
secondary metabolic process: 1.268% (raw value = 6)
post-embryonic development: 1.057% (raw value = 5)
cell communication: 0.846% (raw value = 4)
lipid metabolic process: 0.846% (raw value = 4)
other biological processes: 0.634% (raw value = 3)
growth: 0.634% (raw value = 3)
cell growth: 0.423% (raw value = 2)
cell differentiation: 0.423% (raw value = 2)
translation: 0.211% (raw value = 1)
response to light stimulus: 0.211% (raw value = 1)
generation of precursor metabolites and energy: 0.211% (raw value
tropism: 0.211% (raw value = 1)

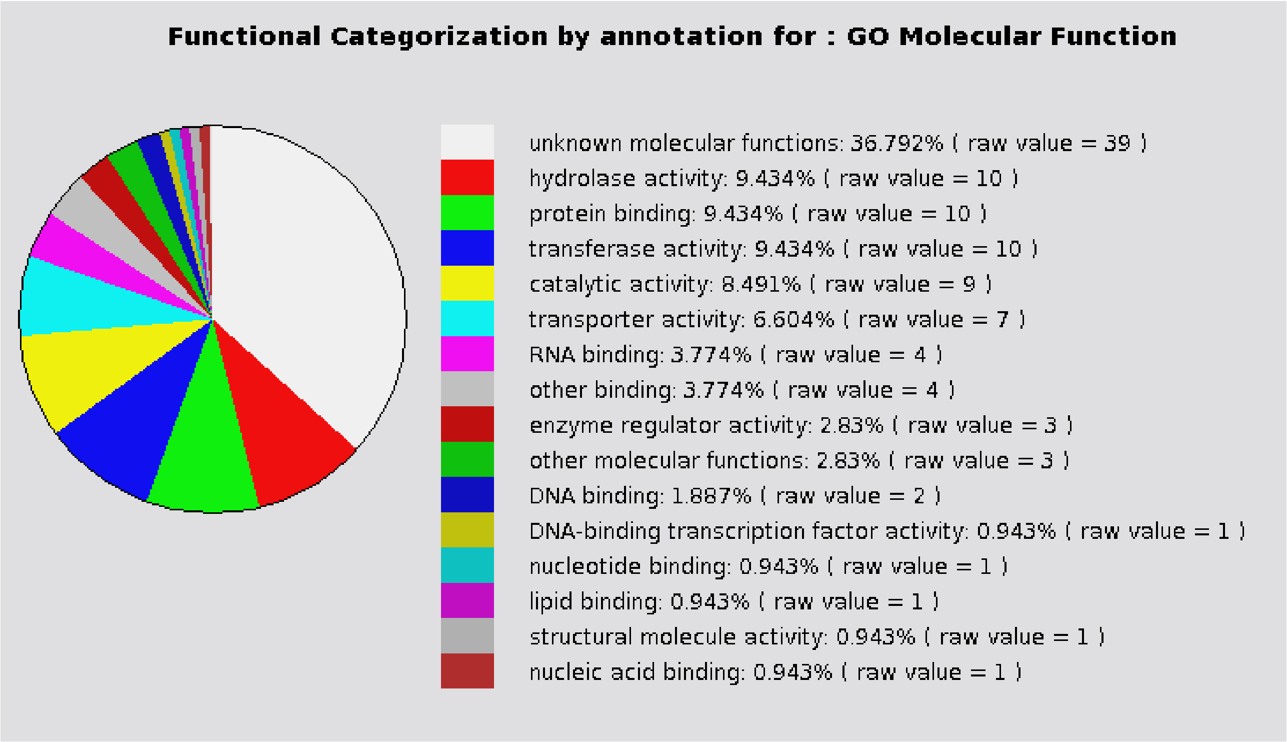
Functional Categorization by annotation for : GO Molecular Function
unknown molecular functions: 36.792% (raw value = 39)
hydrolase activity: 9.434% (raw value = 10)
protein binding: 9.434% (raw value = 10)
transferase activity: 9.434% (raw value = 10)
catalytic activity: 8.491% (raw value = 9)
transporter activity: 6.604% (raw value = 7)
RNA binding: 3.774% (raw value = 4)
other binding: 3.774% (raw value = 4)
enzyme regulator activity: 2.83% (raw value = 3)
other molecular functions: 2.83% (raw value = 3)
DNA binding: 1.887% (raw value = 2)
DNA-binding transcription factor activity: 0.943% (raw value = 1)
nucleotide binding: 0.943% (raw value = 1)
lipid binding: 0.943% (raw value = 1)
structural molecule activity: 0.943% (raw value = 1)
nucleic acid binding: 0.943% (raw value = 1)

DOWNREGULATED GENES

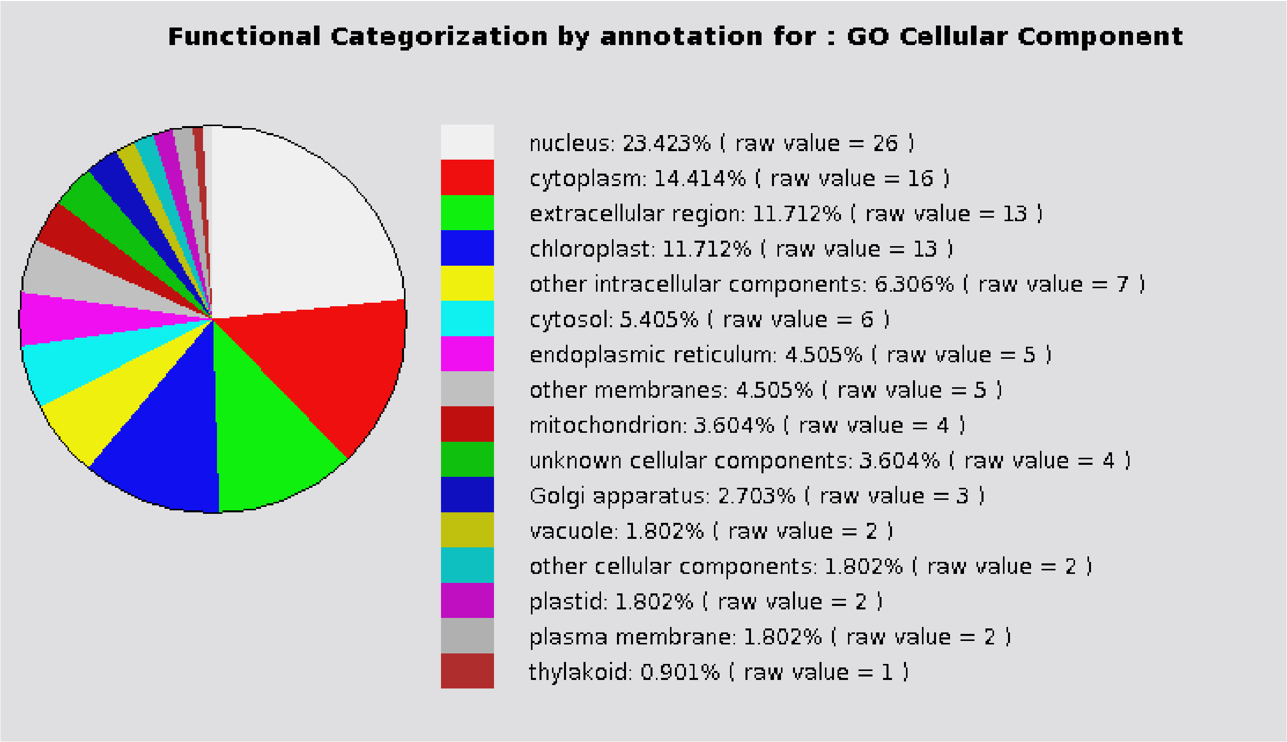

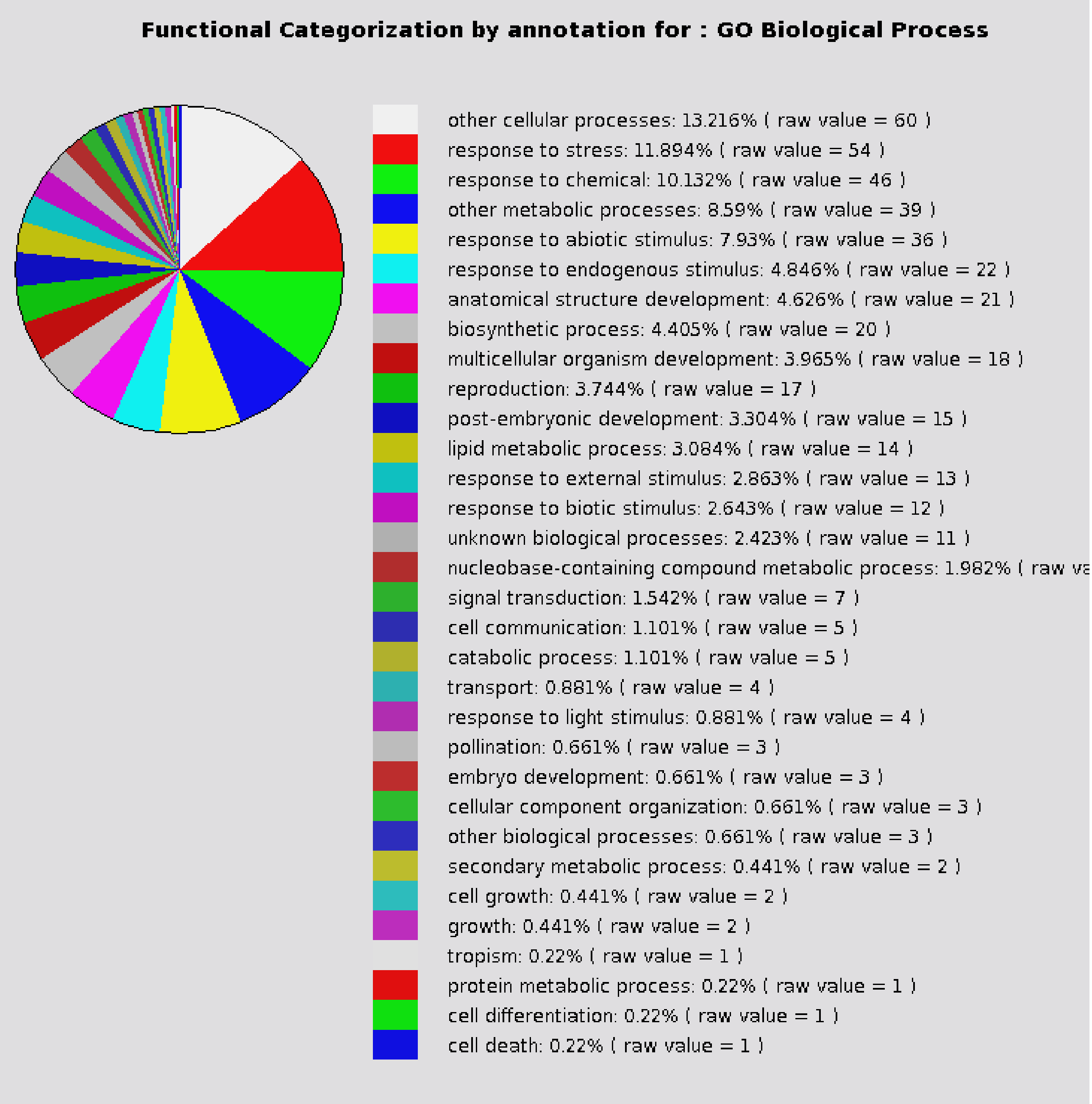
Functional Categorization by annotation for : GO Biological Process
other cellular processes: 13.216% (raw value = 60)
response to stress: 11.894% (raw value = 54)
response to chemical: 10.132% (raw value = 46)
other metabolic processes: 8.59% (raw value = 39)
response to abiotic stimulus: 7.93% (raw value = 36)
response to endogenous stimulus: 4.846% (raw value = 22)
anatomical structure development: 4.626% (raw value = 21)
biosynthetic process: 4.405% (raw value = 20)
multicellular organism development: 3.965% (raw value = 18)
reproduction: 3.744% (raw value = 17)
post-embryonic development: 3.304% (raw value = 15)
lipid metabolic process: 3.084% (raw value = 14)
response to external stimulus: 2.863% (raw value = 13)
response to biotic stimulus: 2.643% (raw value = 12)
unknown biological processes: 2.423% (raw value = 11)
nucleobase-containing compound metabolic process: 1.982% (raw va
signal transduction: 1.542% (raw value = 7)
cell communication: 1.101% (raw value = 5)
catabolic process: 1.101% (raw value = 5)
transport: 0.881% (raw value = 4)
response to light stimulus: 0.881% (raw value = 4)
pollination: 0.661% (raw value = 3)
embryo development: 0.661% (raw value = 3)
cellular component organization: 0.661% (raw value = 3)
other biological processes: 0.661% (raw value = 3)
secondary metabolic process: 0.441% (raw value = 2)
cell growth: 0.441% (raw value = 2)
growth: 0.441% (raw value = 2)
tropism: 0.22% (raw value = 1)
protein metabolic process: 0.22% (raw value = 1)
cell differentiation: 0.22% (raw value = 1)
cell death: 0.22% (raw value = 1)

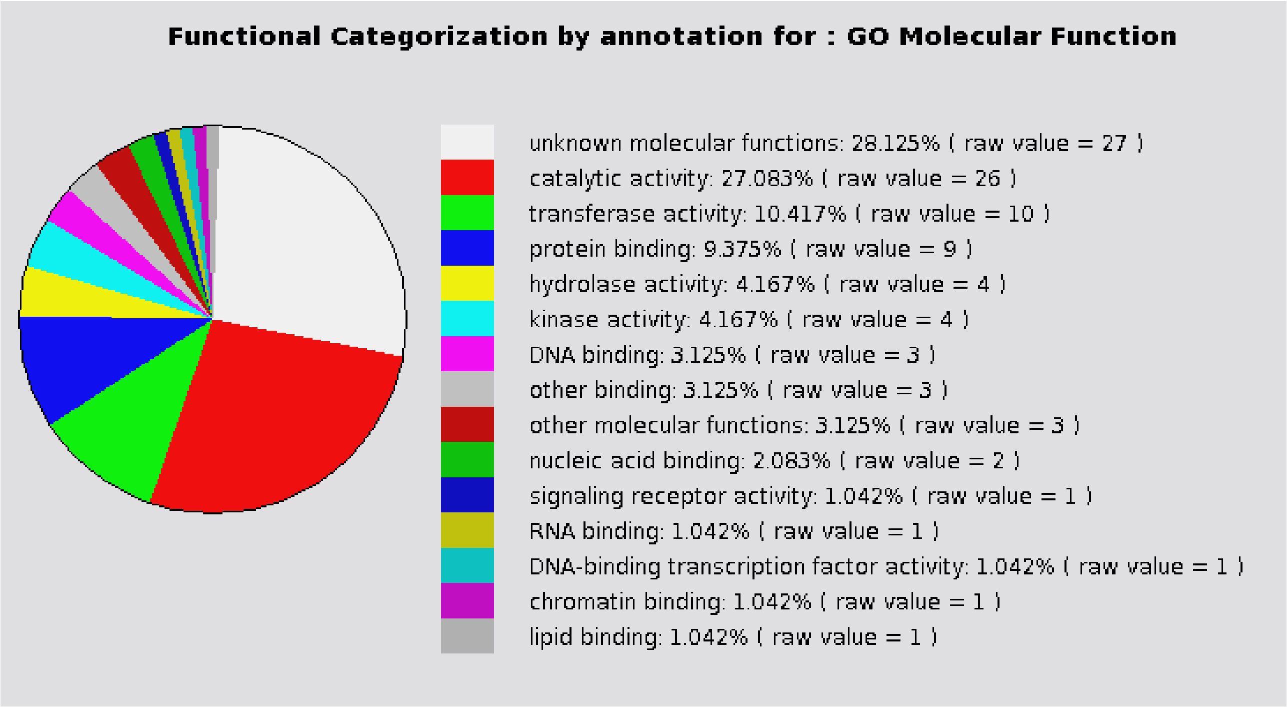
Functional Categorization by annotation for : GO Molecular Function
unknown molecular functions: 28.125% (raw value = 27)
catalytic activity: 27.083% (raw value = 26)
transferase activity: 10.417% (raw value = 10)
protein binding: 9.375% (raw value = 9)
hydrolase activity: 4.167% (raw value = 4)
kinase activity: 4.167% (raw value = 4)
DNA binding: 3.125% (raw value = 3)
other binding: 3.125% (raw value = 3)
other molecular functions: 3.125% (raw value = 3)
nucleic acid binding: 2.083% (raw value = 2)
signaling receptor activity: 1.042% (raw value = 1)
RNA binding: 1.042% (raw value = 1)
DNA-binding transcription factor activity: 1.042% (raw value = 1)
chromatin binding: 1.042% (raw value = 1)
lipid binding: 1.042% (raw value = 1)
