# Supplementary material for: Bacterial Volatiles (mVOC) Emitted by the Phytopathogen Erwinia amylovora Promote Arabidopsis thaliana Growth and Oxidative Stress
Source: Antioxidants (Basel). 2023 Feb 28;12(3):600. doi: 10.3390/antiox12030600 (PMC10045578; doi:10.3390/antiox12030600)
Supplement: Supplementary file 1 [file antioxidants-12-00600-s001.zip › Supplementary Figure S4.pdf]

Supplementary Figure S4

Validation of microarray data was done by selecting three of the most downregulated genes (SESA1, SESA2 and SESA3) which were evaluated in a time-course experiment. Supplementary Figure S3 shows the expression of the three genes from 3 h to 16 h of exposure to E. amylovora MVOCs. A hormetic trend was found for all genes with peaks of expression at 9 and 12 hr (Figure 8)

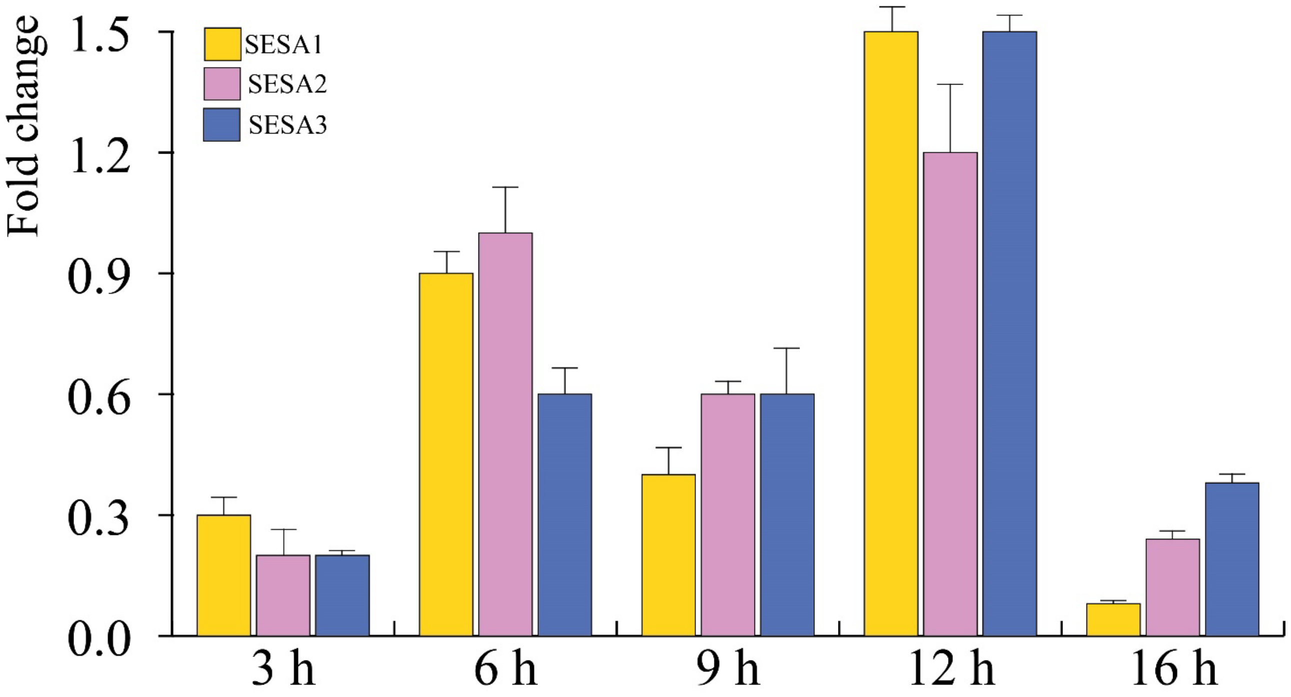

Time course gene expression of A. thaliana SESA1, SESA2 and SESA3 genes after exposure of plants to E. amylovora MVOCs. Data are expressed as fold change with respect to unexposed plants. Metric bars indicate standard deviation.
