# Supplementary material for: Bacterial Volatiles (mVOC) Emitted by the Phytopathogen Erwinia amylovora Promote Arabidopsis thaliana Growth and Oxidative Stress
Source: Antioxidants (Basel). 2023 Feb 28;12(3):600. doi: 10.3390/antiox12030600 (PMC10045578; doi:10.3390/antiox12030600)
Supplement: Supplementary file 1 [file antioxidants-12-00600-s001.zip › Supplementary Figure S5.pdf]

Supplementary Figure S5

DR5::GUS staining

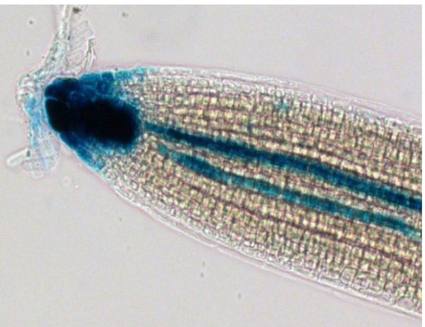

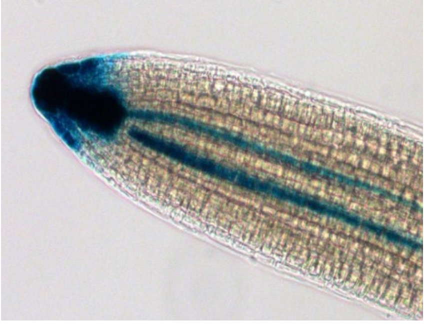

CONTROL

mVOC exposed plants

GFP localization

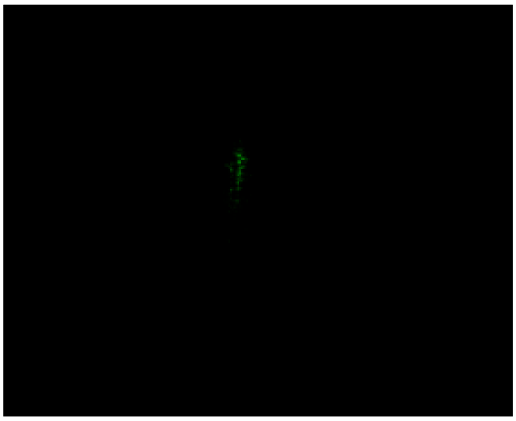

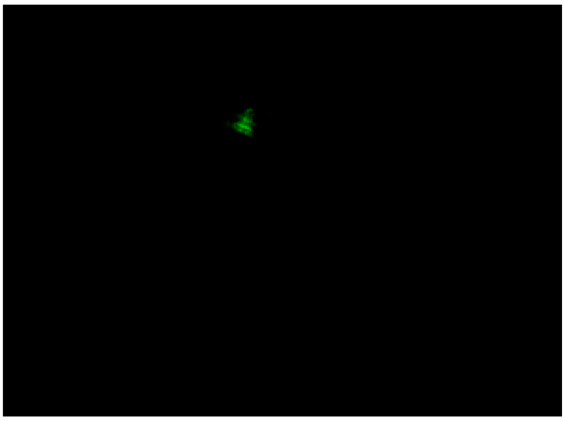

PIN1:GFP CONTROL

PIN3:GFP CONTROL
