# Supplementary material for: Bacterial Volatiles (mVOC) Emitted by the Phytopathogen Erwinia amylovora Promote Arabidopsis thaliana Growth and Oxidative Stress
Source: Antioxidants (Basel). 2023 Feb 28;12(3):600. doi: 10.3390/antiox12030600 (PMC10045578; doi:10.3390/antiox12030600)

Supplementary Figure S7

Density plots of secondary root production in controls and in A. thaliana seedlings exposed to the two synthetic mVOCs. Bars represent the median difference between the number of secondary roots after 72 h with respect to the time 0. X axis indicates the number of secondary roots, Y axis the total counts.

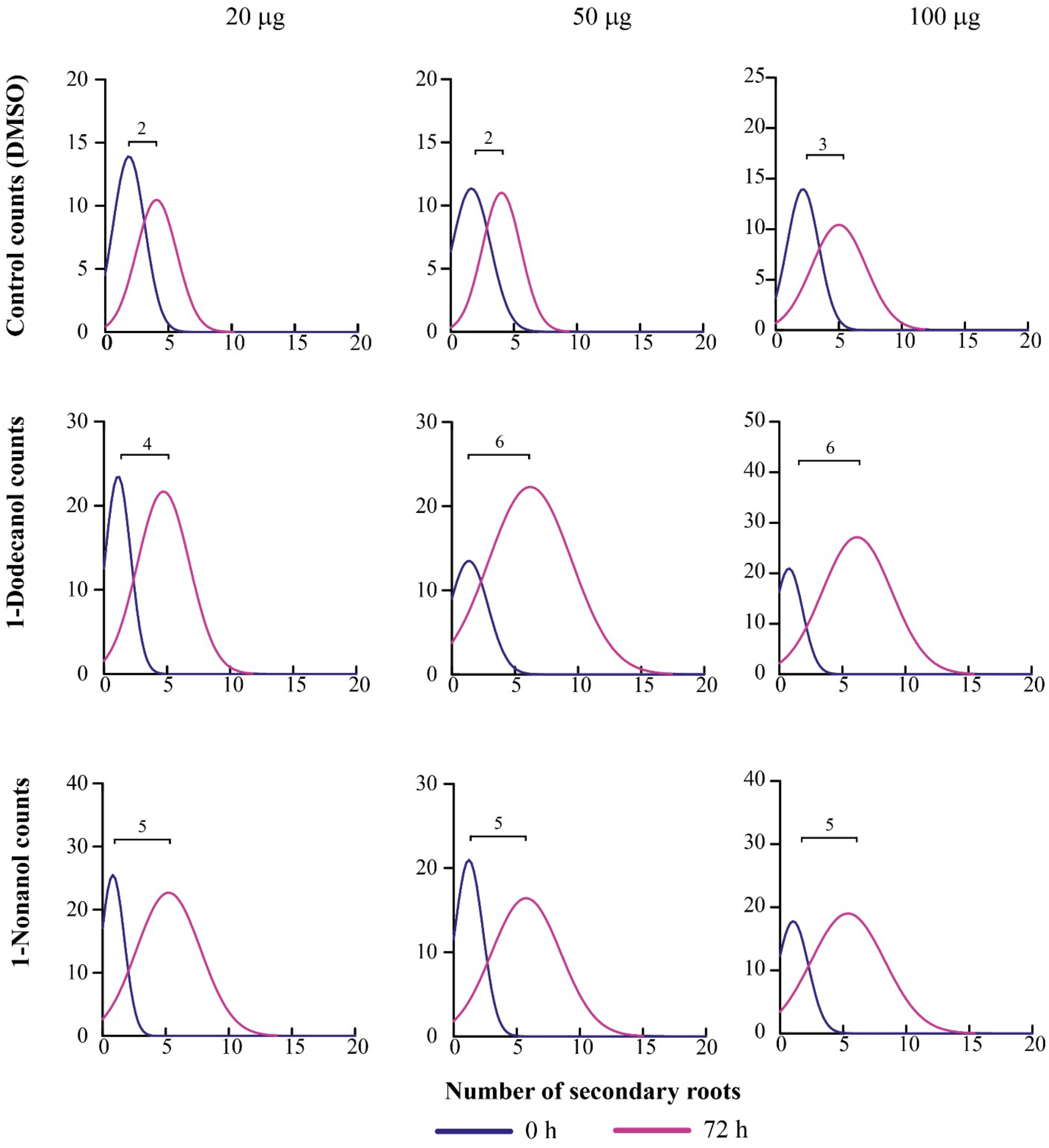

Supplement: Supplementary file 1 [file antioxidants-12-00600-s001.zip › Supplementary Figure S7.pdf]
