# Supplementary material for: Bacterial Volatiles (mVOC) Emitted by the Phytopathogen Erwinia amylovora Promote Arabidopsis thaliana Growth and Oxidative Stress
Source: Antioxidants (Basel). 2023 Feb 28;12(3):600. doi: 10.3390/antiox12030600 (PMC10045578; doi:10.3390/antiox12030600)
Supplement: Supplementary file 1 [file antioxidants-12-00600-s001.zip › Supplementary Table S1.pdf]

Supplementary Table S1: List of primers used in this work.

Gene	Locus	Sequence 5′→3′	
ACT1	*AT2G37620*	TGCACTTCCACATGCTATCC	Forward
ACT1	*AT2G37620*	GAGCTGGTTTTGGCTGTCTC	Reverse
eEF1Balpha2	*AT5G19510*	ACTTGTACCAGTTGGTTATGGG	Forward
eEF1Balpha2	*AT5G19510*	CTGGATGTACTCGTTGTTAGGC	Reverse
SESA1	*AT4G27140*	CATAGGCCCAAAAATGAGGA	Forward
SESA1	*AT4G27140*	GCTGTCCCTGGAGTCTAACG	Reverse
SESA2	*AT4G27150*	TGTCGAGTTCGACGAAGATG	Forward
SESA2	*AT4G27150*	GGCAAACACAAACTGGCTCT	Reverse
SESA3	*AT4G27160*	TGGATGAGCAAGCAAATGAG	Forward
SESA3	*AT4G27160*	AGGGACATTCACCAACTTGC	Reverse
PIN1	*AT1G73590*	ACAAAACGACGCAGGCTAAG	Forward
PIN1	*AT1G73590*	AGGAAATGAGGGACCAGGTG	Reverse
PIN3	*AT1G70940*	GCGCTACAATCCAAGACAGG	Forward
PIN3	*AT1G70940*	GCGACGAGAGCCCAAATAAG	Reverse
CAT1	*AT1G20630*	CAGGACTACAGGCACATGGA	Forward
CAT1	*AT1G20630*	CGACAGGCATTTGATCCCAC	Reverse
SOD1	*AT1G08830*	TGCCACCTTCACAATCACTG	Forward
SOD1	*AT1G08830*	TGAGTTCATGGCCTCCCTTT	Reverse
PER4	*AT1G14540*	GGCTAGCCTCAACGATCTCT	Forward
PER4	*AT1G14540*	GCGTCAATGTCACTCGAGTT	Reverse
APX1	*AT1G07890*	GGGTCGTCTTCCTGATGCTA	Forward
APX1	*AT1G07890*	GGGTCGTCTTCCTGATGCTA	Reverse
RBOHH	*AT5G60010*	TTAGTGGTGGAGGAGGCAAG	Forward
RBOHH	*AT5G60010*	TGCATCTCCAGCTTCGTACA	Reverse
