# Supplementary material for: Bacterial Volatiles (mVOC) Emitted by the Phytopathogen Erwinia amylovora Promote Arabidopsis thaliana Growth and Oxidative Stress
Source: Antioxidants (Basel). 2023 Feb 28;12(3):600. doi: 10.3390/antiox12030600 (PMC10045578; doi:10.3390/antiox12030600)
Supplement: Supplementary file 1 [file antioxidants-12-00600-s001.zip › Supplementary Table S2.pdf]

Supplementary Table S2. Differentially expressed genes in seedlings of *A. thaliana* exposed to *E. amylovora* mVOCs. Values are expressed as fold change with re-spect to unexposed plants.

Locus tag	Gene symbol	Fold change	Gene description	Gene Ontology		
				**Molecular function**	**Biological process**	**Cellular component**
**UPREGULATED GENES**						
AT1G03103	*AT1G03103*	2.05	bifunctional inhibitor/lipid-transfer protein/seed storage 2S albumin superfamily protein			extracellular region
AT1G06340	*AT1G06340*	3.80	plant Tudor-like protein			
AT1G07645	*DSI-1VOC*	2.04	desiccation-induced 1VOC-like protein			extracellular region
AT1G09860	*PUP16*	2.27	purine permease 16	purine nucleobase transmembrane transporter activity	purine nucleobase transport	membrane
AT1G09935	*AT1G09935*	2.45	Phosphoglycerate mutase family protein	catalytic activity	biological process	
AT1G09950	*RAS1*	2.94	response to ABA and salt 1			
AT1G12805	*AT1G12805*	2.49	nucleotide binding protein			mitochondrion
AT1G14880	*PCR1*	2.47	cadmium resistance protein 1	mRNA binding	defense response to bacterium	cytoplasm
AT1G15520	*ABCG40*	2.57	pleiotropic drug resistance 12	efflux transmembrane transporter activity	abscisic acid transport	mitochondrion
AT1G21990	*AT1G21990*	4.93	F-box/RNI-like/FBD-like domains-containing protein			
AT1G27461	*AT1G27461*	2.88	uncharacterized protein	core promoter sequence-specific DNA binding	seed dormancy process	cytosol
AT1G28260	*AT1G28260*	2.10	Telomerase activating protein Est1			
AT1G30950	*UFO*	2.41	UNUSUAL FLORAL ORGANS F-box family protein	transcription factor binding	SCF-dependent proteasomal ubiquitin-	part of ubiquitin ligase complex

Locus tag	Gene symbol	Fold change	Gene description	Gene Ontology		
				**Molecular function**	**Biological process**	**Cellular component**
					dependent protein catabolic process	
AT1G50060	*AT1G50060*	2.80	CAP (Cysteine-rich secretory proteins, Antigen 5, and Pathogenesis-related 1 protein) superfamily protein			extracellular region
AT1G54020	*AT1G54020*	2.60	GDSL-like Lipase/Acyl hydrolase superfamily protein			
AT1G57943	*PUP17*	4.38	purine permease 17	purine nucleobase transmembrane transporter activity	purine nucleobase transport	membrane
AT1G60570	*AT1G60570*	2.06	Galactose oxidase/kelch repeat superfamily protein			cytoplasm
AT1G64130	*AT1G64130*	2.23	polyketide cyclase/dehydrase and lipid transport superfamily protein		macromolecule biosynthetic process	
AT1G64160	*DIR5*	4.24	Disease resistance-responsive (dirigent-like protein) family protein	guiding stereospecific synthesis activity	(-)-pinoresinol biosynthetic process	extracellular region
AT1G65570	*AT1G65570*	2.14	Pectin lyase-like superfamily protein	polygalacturonase activity	carbohydrate metabolic process	apoplast
AT1G69818	*AT1G69818*	2.05	defensin-like protein			extracellular region
AT1G69920	*GSTU12*	2.08	glutathione S-transferase TAU 12	glutathione transferase activity	toxin catabolic process	cytoplasm
AT1G72260	*THI2.1*	4.35	thionin 2.1	toxin activity	jasmonic acid mediated signaling pathway	extracellular region
AT1G77655	*AT1G77655*	2.55	uncharacterized protein		cellular component assembly	mitochondrion

Locus tag	Gene symbol	Fold change	Gene description	Gene Ontology		
				Molecular function	Biological process	Cellular component
AT1G79800	*ENODL7*	3.58	early nodulin-like protein 7	electron transfer activity		membrane
AT2G02620	*AT2G02620*	2.80	cysteine/histidine-rich C1 domain-containing protein			nucleus
AT2G03460	*AT2G03460*	2.97	Galactose oxidase/kelch repeat superfamily protein			
AT2G04045	*AT2G04045*	2.71	Defensin-like (DEFL) family protein			extracellular region
AT2G11005	*AT2G11005*	2.02	glycine-rich protein			nucleus
AT2G14378	*AT2G14378*	2.48	egg cell-secreted-like protein (DUF1278)			
AT2G14610	*PR1*	2.10	pathogenesis-related protein 1		defense response	extracellular region
AT2G19550	*AT2G19550*	2.30	alpha/beta-Hydrolases superfamily protein			membrane
AT2G22060	*AT2G22060*	2.62	galactose oxidase/kelch repeat protein			mitochondrion
AT2G22460	*AT2G22460*	2.03	MIZU-KUSSEI-like protein		hydrotropism	nucleus
AT2G25685	*SCRL17*	2.15	SCR-like protein 17			
AT2G25890	*AT2G25890*	2.06	Oleosin-like protein			mitochondrion
AT2G26130	*RSL1*	2.46	RING/U-box protein with C6HC-type zinc finger	RNA-DNA hybrid ribonuclease activity	gibberellin mediated signaling pathway	nucleus
AT2G26450	*AT2G26450*	2.06	Plant invertase/pectin methylesterase inhibitor superfamily		cell differentiation	
AT2G29470	*GSTU3*	5.19	glutathione S-transferase TAU 3		toxin catabolic process	cytoplasm
AT2G30770	*CYP71A13*	4.16	cytochrome P450, family 71, subfamily A, polypeptide 13	indoleacetaldoxime dehydratase activity	camalexin biosynthetic process	endoplasmic reticulum lumen

Locus tag	Gene symbol	Fold change	Gene description	Gene Ontology		
				Molecular function	Biological process	Cellular component
AT2G32275	*AT2G32275*	2.07	uncharacterized protein			nucleus
AT2G33720	*AT2G33720*	5.77	AP2/B3-like transcriptional factor family protein	DNA-binding transcription factor activity	response to lipid	nucleus
AT2G34090	*MEE18*	2.02	maternal effect embryo arrest 18 protein	LPPG:FO 2-phospho-L-lactate transferase activity	embryo development ending in seed dormancy	mitochondrion
AT2G38240	*JAO4*	2.61	2-oxoglutarate (2OG) and Fe(II)-dependent oxygenase-like protein	jasmonic acid hydrolase	defense response to fungus	cytoplasm
AT2G44240	*AT2G44240*	4.85	NEP-interacting protein (DUF239)		response to oxidative stress	extracellular region
AT3G10300	*AT3G10300*	2.06	Calcium-binding EF-hand family protein	calcium ion binding	hormone-mediated signaling pathway	nucleus
AT3G14415	*GOX2*	2.61	Aldolase-type TIM barrel family protein	(S)-2-hydroxy-acid oxidase activity	defense response to bacterium	apoplast
AT3G21500	*DXS2*	3.31	1-deoxy-D-xylulose 5-phosphate synthase 1	NOT 1-deoxy-D-xylulose-5-phosphate synthase activity	terpenoid biosynthetic process	chloroplast
AT3G23420	*AT3G23420*	3.12	F-box and associated interaction domain-containing protein			
AT3G27503	*SCRL19*	2.24	SCR-like 19		signal transduction	extracellular region
AT3G28220	*AT3G28220*	2.97	TRAF-like family protein		alpha-amino acid metabolic process	chloroplast envelope
AT3G30720	*QQS*	2.56	qua-quine starch	protein binding	negative regulation of starch metabolic process	cytosol
AT3G43572	*AT3G43572*	2.42	uncharacterized protein			
AT3G43850	*AT3G43850*	3.05	uncharacterized protein		defense response to bacterium	nucleus

Locus tag	Gene symbol	Fold change	Gene description	Gene Ontology		
				**Molecular function**	**Biological process**	**Cellular component**
AT3G49840	*AT3G49840*	3.15	uncharacterized transmembrane protein		chromatin organization	nuclear membrane
AT3G55970	*JOX3*	3.72	jasmonate-regulated protein JRG21	jasmonic acid hydrolase	defense response to fungus	cytoplasm
AT3G60120	*BGLU27*	2.93	beta glucosidase 27	scopolin beta-glucosidase activity	carbohydrate metabolic process	nucleus
AT3G62950	*AT3G62950*	2.61	Thioredoxin superfamily protein	protein binding	negative regulation of transcription by RNA polymerase II	cytoplasm
AT4G10265	*AT4G10265*	4.76	Wound-responsive family protein		cellular response to hypoxia	nucleus
AT4G11911	*AT4G11911*	2.53	STAY-GREEN-like protein			
AT4G14315	*AT4G14315*	3.35	uncharacterized protein		regulation of developmental process	
AT4G16590	*CSLA01*	3.37	cellulose synthase-like A01	glycosyltransferase activity	anthocyanin-containing compound metabolic process	Golgi apparatus
AT4G22650	*AT4G22650*	4.88	lipid transfer protein			extracellular region
AT4G23520	*AT4G23520*	3.30	Cysteine proteinases superfamily protein			
AT4G26390	*AT4G26390*	2.20	Pyruvate kinase family protein	magnesium ion binding	glycolytic process	cytoplasm
AT4G26880	*AT4G26880*	2.41	Stigma-specific Stig1 family protein		tissue development	extracellular region
AT4G30064	*LCR61*	2.25	low-molecular-weight cysteine-rich 61		defense response to bacterium	extracellular region
AT4G33230	*AT4G33230*	2.43	Plant invertase/pectin methylesterase inhibitor superfamily		cell wall modification	

Locus tag	Gene symbol	Fold change	Gene description	Gene Ontology		
				Molecular function	Biological process	Cellular component
AT4G39750	*AT4G39750*	2.19	F-box family protein-like protein			nucleus
AT5G05290	*EXPA2*	2.52	expansin A2		plant-type cell wall loosening	extracellular region
AT5G07510	*GRP14*	2.77	glycine-rich protein 14	lipid binding	sexual reproduction	extracellular region
AT5G14950	*GMII*	2.14	Golgi alpha-mannosidase II	alpha-mannosidase activity	hyperosmotic salinity response	Golgi apparatus
AT5G17100	*AT5G17100*	2.69	Cystatin/monellin superfamily protein			nucleus
AT5G18748	*AT5G18748*	2.35	uncharacterized protein			mitochondrion
AT5G18880	*AT5G18880*	3.65	RNA-directed DNA polymerase (reverse transcriptase)-related family protein			
AT5G22610	*AT5G22610*	2.89	F-box/RNI-like/FBD-like domains-containing protein			
AT5G25425	*AT5G25425*	3.96	glycine-rich protein			
AT5G33390	*AT5G33390*	2.61	glycine-rich protein			
AT5G36970	*NHL25*	2.81	NDR1/HIN1-like 25			
AT5G37640	*UBQ9*	4.24	ubiquitin 9	mRNA binding	ubiquitin-dependent protein catabolic process	nucleolus
AT5G38270	*AT5G38270*	2.47	F-box family protein			nucleus
AT5G39650	*DAU2*	7.08	DAU2 transmembrane protein, putative (DUF679)	molecular carrier activity	double fertilization forming a zygote and endosperm	endoplasmic reticulum lumen
AT5G53260	*AT5G53260*	2.29	Seed maturation protein			cytosol
AT5G63070	*AT5G63070*	2.29	Ribosomal protein S19 family protein	RNA binding	translation	cytoplasm
**DOWNREGULATED GENES**						

Locus tag	Gene symbol	Fold change	Gene description	Gene Ontology		
				Molecular function	Biological process	Cellular component
AT1G03880	*CRU2*	0.20	cruciferin 2	nutrient reservoir activity	cellular response to abscisic acid stimulus	
AT1G06280	*LBD2*	0.34	LOB domain-containing protein 2			nucleus
AT1G07430	*HAI2*	0.49	highly ABA-induced PP2C protein 2	myosin phosphatase activity	negative regulation of abscisic acid-activated signaling pathway	chloroplast
AT1G09080	*BIP3*	0.49	Heat shock protein 70 (Hsp 70) family protein	ATP-dependent protein folding chaperone	pollen tube growth	chloroplast
AT1G09510	*AT1G09510*	0.15	NAD(P)-binding Rossmann-fold superfamily protein	alcohol dehydrogenase (NAD+) activity		
AT1G18350	*MKK7*	0.45	MAP kinase kinase 7	kinase activity	auxin polar transport	
AT1G43890	*RAB18*	0.23	RAB GTPASE HOMOLOG B18			
AT1G45223	*AT1G45223*	0.35	ECA1 gametogenesis related family protein			extracellular region
AT1G52690	*LEA7*	0.31	Late embryogenesis abundant protein (LEA) family protein		protein stabilization	cytosol
AT1G57760	*AT1G57760*	0.31	ECA1 gametogenesis related family protein			extracellular region
AT1G60190	*PUB19*	0.27	ARM repeat superfamily protein	ubiquitin-protein transferase activity	regulation of seed germination	chloroplast
AT1G68250	*AT1G68250*	0.18	uncharacterized protein			
AT1G71000	*AT1G71000*	0.44	Chaperone DnaJ-domain superfamily protein		defense response to fungus	cytoplasm
AT1G72980	*LBD7*	0.50	LOB domain-containing protein 7			nucleus

Locus tag	Gene symbol	Fold change	Gene description	Gene Ontology		
				Molecular function	Biological process	Cellular component
AT1G80260	*emb1427*	0.49	Spc97 / Spc98 family of spindle pole body (SBP) component	gamma-tubulin binding	embryo development ending in seed dormancy	microtubule organizing center
AT2G02700	*AT2G02700*	0.30	cysteine/histidine-rich C1 domain-containing protein			nucleus
AT2G03090	*EXPA15*	0.40	expansin A15		plant-type cell wall loosening	extracellular region
AT2G15010	*AT2G15010*	0.24	Plant thionin		defense response	extracellular region
AT2G18340	*AT2G18340*	0.44	late embryogenesis abundant domain-containing protein		response to water deprivation	endoplasmic reticulum
AT2G26150	*HSFA2*	0.48	heat stress transcription factor A2	DNA-binding transcription factor activity	cellular response to hypoxia	nucleus
AT2G28090	*AT2G28090*	0.48	Heavy metal transport/detoxification superfamily protein			nucleus
AT2G28860	*CYP710A4*	0.32	cytochrome P450 710A4	C-22 sterol desaturase activity	sterol biosynthetic process	extracellular region
AT2G29380	*HAI3*	0.42	highly ABA-induced PP2C protein 3	myosin phosphatase activity		nucleus
AT2G34370	*AT2G34370*	0.38	Pentatricopeptide repeat (PPR) superfamily protein	zinc ion binding		mitochondrion
AT2G42000	*AtMT4a*	0.29	plant EC metallothionein family protein	zinc ion binding	zinc ion transport	cytoplasm
AT2G42560	*AT2G42560*	0.24	late embryogenesis abundant domain-containing protein		cold acclimation	cytosol
AT2G47770	*TSPO*	0.46	outer membrane tryptophan-rich sensory protein-like protein	heme binding	response to abscisic acid	Golgi apparatus
AT3G01326	*AT3G01326*	0.39	ECA1 gametogenesis related family protein			

Locus tag	Gene symbol	Fold change	Gene description	Gene Ontology		
				Molecular function	Biological process	Cellular component
AT3G02480	*AT3G02480*	0.35	Late embryogenesis abundant protein (LEA) family protein		negative regulation of leaf senescence	cytosol
AT3G11480	*BSMT1*	0.30	S-adenosyl-L-methionine-dependent methyltransferases superfamily protein	S-adenosyl-L-methionine:benzoic acid carboxyl methyl transferase activity	defense response	nucleus
AT3G14440	*NCED3*	0.49	9-cis-epoxycarotenoid dioxygenase 3	9-cis-epoxycarotenoid dioxygenase activity	abscisic acid biosynthetic process	chloroplast
AT3G17520	*AT3G17520*	0.42	late embryogenesis abundant protein (LEA) family protein		acquisition of seed longevity	endoplasmic reticulum
AT3G20160	*GGPPS8*	0.40	Terpenoid synthases superfamily protein	dimethylallyltranstransferase activity	farnesyl diphosphate biosynthetic process	chloroplast
AT3G22640	*PAP85*	0.37	cupin family protein		response to virus	extracellular region
AT3G25810	*AT3G25810*	0.39	Terpenoid cyclases/Protein prenyltransferases superfamily protein	(4S)-limonene synthase activity	monoterpene biosynthetic process	chloroplast
AT3G27025	*AT3G27025*	0.48	uncharacterized protein		regulation of auxin polar transport	chloroplast
AT3G49620	*DIN11*	0.47	2-oxoglutarate (2OG) and Fe(II)-dependent oxygenase superfamily protein		cellular response to starvation	cytoplasm
AT3G54490	*RPB5E*	0.34	RNA polymerase II fifth largest subunit, E	DNA binding	DNA-templated transcription	nucleus
AT3G58390	*AT3G58390*	0.26	Eukaryotic release factor 1 (eRF1) family protein	protein binding	nuclear-transcribed mRNA catabolic process	nucleus

Locus tag	Gene symbol	Fold change	Gene description	Gene Ontology		
				Molecular function	Biological process	Cellular component
AT4G03480	*AT4G03480*	0.31	Ankyrin repeat family protein		defense response to bacterium	chloroplast
AT4G09600	*GASA3*	0.32	GAST1 protein homolog 3		response to gibberellin	extracellular region
AT4G10250	*ATHSP22.0*	0.40	HSP20-like chaperones superfamily protein		cellular response to hypoxia	chloroplast
AT4G27140	*SESA1*	0.07	seed storage albumin 1		defense response	extracellular region
AT4G27150	*SESA2*	0.12	seed storage albumin 2			extracellular region
AT4G27160	*SESA3*	0.30	seed storage albumin 3	protein histidine kinase binding	seed maturation	extracellular region
AT4G28520	*CRU3*	0.09	cruciferin 3	nutrient reservoir activity	cellular response to abscisic acid stimulus	protein body
AT4G33467	*AT4G33467*	0.20	uncharacterized protein		response to abscisic acid	nucleus
AT4G33905	*AT4G33905*	0.47	Peroxisomal membrane 22 kDa		response to abscisic acid	mitochondrion
AT4G37990	*ELI3-2*	0.47	cinnamyl alcohol dehydrogenase 8	aryl-alcohol dehydrogenase (NADP+) activity	lignin biosynthetic process	cytoplasm
AT5G03210	*DIP2*	0.42	E3 ubiquitin-protein ligase		defense response to virus	nucleus
AT5G04120	*AT5G04120*	0.41	Phosphoglycerate mutase family protein	L-phosphoserine phosphatase activity	D-serine biosynthetic process	cytoplasm
AT5G04380	*AT5G04380*	0.17	S-adenosyl-L-methionine-dependent methyltransferases superfamily protein			nucleus

Locus tag	Gene symbol	Fold change	Gene description	Gene Ontology		
				Molecular function	Biological process	Cellular component
AT5G05220	*AT5G05220*	0.15	uncharacterized protein		abscisic acid-activated signaling pathway	chloroplast
AT5G06760	*LEA4-5*	0.23	late embryogenesis abundant protein 4-5	osmosensor activity	response to osmotic stress	cytosol
AT5G07260	*AT5G07260*	0.48	START (StAR-related lipid-transfer) lipid-binding domain-containing protein	lipid binding	somatic embryogenesis	nucleus
AT5G24080	*AT5G24080*	0.49	Protein kinase superfamily protein	protein serine/threonine kinase activity	response to abscisic acid	extracellular region
AT5G27260	*AT5G27260*	0.47	Myb/SANT-like DNA-binding domain protein			nucleus
AT5G34905	*AT5G34905*	0.41	ECA1 gametogenesis family protein			chloroplast
AT5G35660	*AT5G35660*	0.39	glycine-rich protein			
AT5G40790	*AT5G40790*	0.35	uncharacterized protein		negative regulation of DNA-templated transcription initiation	nucleus
AT5G42797	*LCR28*	0.39	low-molecular-weight cysteine-rich 28			
AT5G43840	*HSFA6A*	0.48	heat stress transcription factor A6A	DNA-binding transcription factor activity	regulation of DNA-templated transcription	nucleus
AT5G45630	*AT5G45630*	0.40	senescence regulator		response to abscisic acid	cytoplasm
AT5G50360	*AT5G50360*	0.27	von Willebrand factor A domain protein		negative regulation of DNA-templated transcription initiation	nucleus

Locus tag	Gene symbol	Fold change	Gene description	Gene Ontology		
				**Molecular function**	**Biological process**	**Cellular component**
AT5G50600	*HSD1*	0.28	hydroxysteroid dehydrogenase 1	11-beta-hydroxysteroid dehydrogenase (NADP+) activity	response to abscisic acid	chloroplast
AT5G52300	*LTI65*	0.36	low-temperature-induced 65		response to abscisic acid	cytoplasm
AT5G53710	*AT5G53710*	0.49	uncharacterized protein		response to abscisic acid	mitochondrion
AT5G53905	*AT5G53905*	0.43	prolamin-like protein		macromolecule biosynthetic process	nucleus
AT5G54740	*SESA5*	0.02	seed storage albumin 5		pollen development	extracellular region
AT5G65800	*ACS5*	0.43	1-aminocyclopropane-1-carboxylate synthase 5	1-aminocyclopropane-1-carboxylate synthase activity	ethylene biosynthetic process	cytoplasm
